# Hypergravity-Induced Accumulation: A New, Efficient, and Simple Strategy to Improve the Thermal Conductivity of Boron Nitride Filled Polymer Composites

**DOI:** 10.3390/polym13030459

**Published:** 2021-01-31

**Authors:** Kangkang Yu, Tao Yuan, Songdi Zhang, Chenlu Bao

**Affiliations:** 1 School of Materials Science and Engineering, Tiangong University, 399 Binshui West Road, Tianjin 300387, China; yu_kangkang@qq.com (K.Y.); 2515801780@qq.com (T.Y.); sondy790258857@qq.com (S.Z.); 2 Tianjin HaiTe Thermal Management Technology Co., Ltd., 6 Huake 8 Road, Tianjin 300450, China

**Keywords:** boron nitride, silicone rubber, hypergravity, thermal conductivity, microstructure, high viscosity problem, MPPT theory, thermal conductive polymer composites, fillers

## Abstract

Thermal conductive polymer composites (filled type) consisting of thermal conductive fillers and a polymer matrix have been widely used in a range of areas. More than 10 strategies have been developed to improve the thermal conductivity of polymer composites. Here we report a new “hypergravity accumulation” strategy. Raw material mixtures of boron nitride/silicone rubber composites were treated in hypergravity fields (800–20,000 g, relative gravity acceleration) before heat-curing. A series of comparison studies were made. It was found that hypergravity treatments could efficiently improve the microstructures and thermal conductivity of the composites. When the hypergravity was about 20,000 g (relative gravity acceleration), the obtained spherical boron nitride/silicone rubber composites had highly compacted microstructures and high and isotropic thermal conductivity. The highest thermal conductivity reached 4.0 W/mK. Thermal interface application study showed that the composites could help to decrease the temperature on a light-emitting diode (LED) chip by 5 °C. The mechanism of the improved microstructure increased thermal conductivity, and the high viscosity problem in the preparation of boron nitride/silicone rubber composites, and the advantages and disadvantages of the hypergravity accumulation strategy, were discussed. Overall, this work has provided a new, efficient, and simple strategy to improve the thermal conductivity of boron nitride/silicone rubber and other polymer composites (filled type).

## 1. Introduction

With increasing integration and progressive miniaturization, electronic and energy storage devices need to dissipate heat with a high efficiency [1,2,3]. Thermal conductive polymer composites (filled type) composed of a polymer matrix and thermal conductive fillers play an important role in the cooling systems in these areas. These composites are usually prepared by dispersing thermal conductive fillers in a polymer matrix with the techniques of in-situ polymerization, melting blending, and solvent blending, etc. [4,5,6]. They can be used in various ways such as for thermal interface materials, thermal conducting substrates, heat sinks, thermal conductive membranes, and thermal conducting shells. Their practical applications cover a wide range of fields, including mobile phones, computers, appliances, electrical adaptors, batteries, transformers, base stations, and light-emitting diodes (LED), etc. [7,8,9].

Thermal conductivity is the decisive factor affecting the service performance of thermal conductive polymer composites. High thermal conductivity is welcome because it speeds up heat spread. The thermal conductivity is influenced by a range of factors, including thermal conductivity of polymeric matrix, thermal conductivity of fillers, filler concentration, the shape and size of fillers, filler–polymer interface, orientation of fillers, and synergistic effect between different fillers.

In recent years, great efforts have been made to improve the thermal conductivity of polymer composites. A variety of strategies have been developed. The most widely studied strategies include: (i) using a highly thermal conductive polymeric matrix such as nylon, liquid crystal polymers, or ultrahigh molecular weight polyethylene [10,11,12]; (ii) using highly thermal conductive fillers such as graphene, carbon nanotubes (CNTs), boron nitride (BN), and silver nanowires [13,14,15]; (iii) increasing filler concentration (volume fraction or mass fraction); (iv) dispersing thermal conductive fillers in a homogenous state [16,17,18]; (v) exfoliating two-dimensional (2D) thermal conductive fillers such as graphene and BN [19,20,21]; (vi) surface modification of thermal conductive fillers [22,23,24]; (vii) orientating thermal conductive fillers along one direction to promote the thermal conductivity in this direction [25,26,27]; (viii) making thermal conductive fillers into 3-dimensional (3D) porous structures which act as thermal conductive skeleton in the composites [28,29,30]; (ix) using two or more kinds of thermal conductive fillers or using one kind of filler with different sizes to obtain synergism; and (x) constructing thermal conductive pathways in the polymer matrix with templates [31,32,33]. These strategies can be used alone, or in combination. A combination of various strategies can obtain higher thermal conductivity which is not available in normal composites. For example, by using highly crystalline graphite fibers (thermal conductive in axial direction: 600–900 W/mK) and aligning the fibers in alumina/silicone rubber (SR) mixtures with the assistance of a magnetic field, the resultant graphite fibers/alumina/silicone rubber composites can reach very high thermal conductivity (>15 W/mK) at the through-plane direction and meanwhile maintain electrical insulation (no academic reference, several companies such as Fujipoly and Henkel can provide such products).

**Figure 1:**
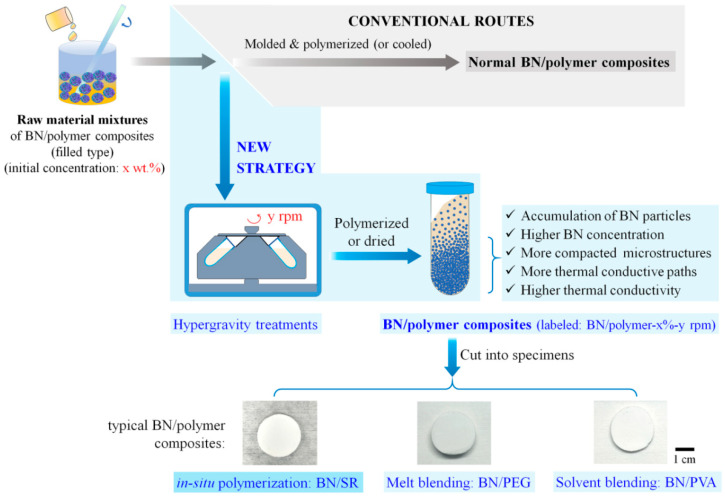
Diagram of the hypergravity accumulation strategy. This new strategy can efficiently improve the thermal conductivity of boron nitride (BN)-filled polymer composites. It is a universal method to be used in different polymers and in various preparation techniques.

**Figure 2:**
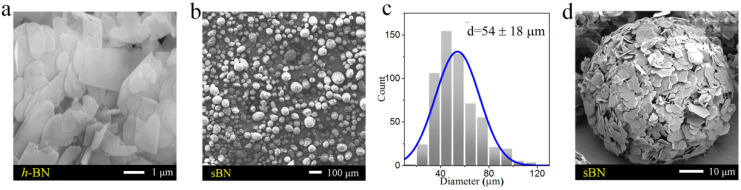
The morphology of BN. (**a**) SEM image of *h-*BN. (**b**) SEM image of spherical assembled *h-*BN (sBN). (**c**) Size statistic diagram of the sBN in (**b**). (**d**) SEM image showing that sBN consists of *h-*BN sheets.

**Figure 3:**
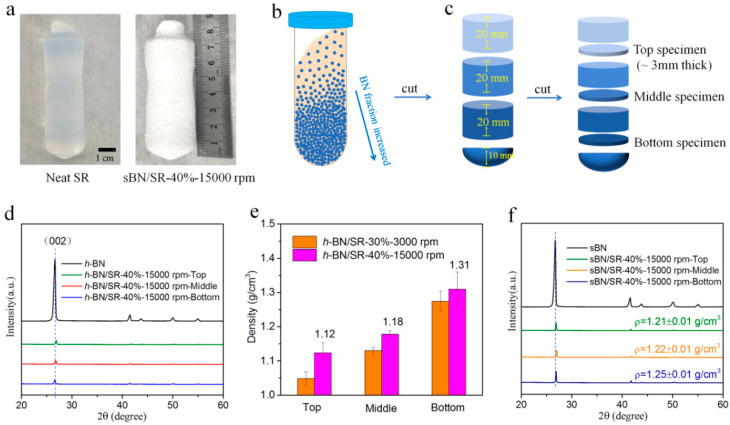
Hypergravity-induced accumulation of BN. (**a**) Photograph of neat silicone rubber and sBN/silicone rubber (SR) composite prepared with hypergravity treatments. (**b**) Scheme diagram of the accumulation of BN. (**c**) Scheme diagram of cutting specimens from different parts of the composites. (**d**) XRD patterns of *h-*BN and *h-*BN/SR composites. (**e**) The density of *h-*BN/SR composites in different parts. (**f**) XRD patterns of sBN and sBN/SR composites. The bottom specimens have stronger *h-*BN’s (002) diffraction peaks and higher density than others, suggesting that hypergravity treatments lead to accumulation of BN in the bottom position of the composites.

**Figure 4:**
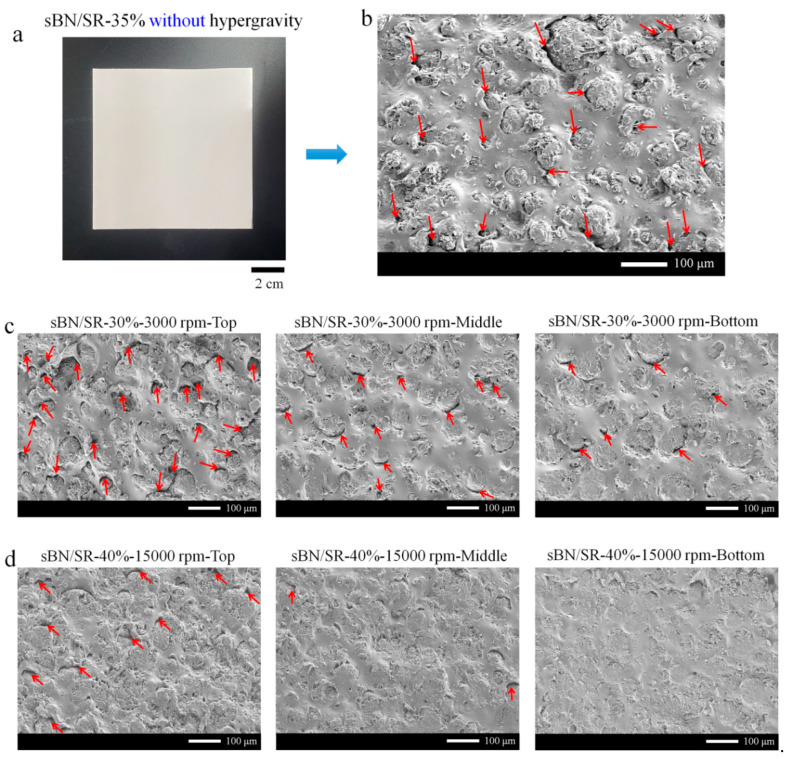
The microstructures of sBN/SR composites. (**a**) Photograph of sBN/SR composite prepared by mechanical mixing and molding (without hypergravity treatments). (**b**) SEM images of the cross-section of sBN/SR composites shown in (**a**). (**c**,**d**) SEM images of the cross-sections of sBN/SR composites prepared with hypergravity treatments. The red arrows refer to micro-cracks. It is clear that hypergravity treatments can remove micro-cracks and bring more compacted microstructures to the composites. Scale bars in SEM images: 100 µm.

**Figure 5:**
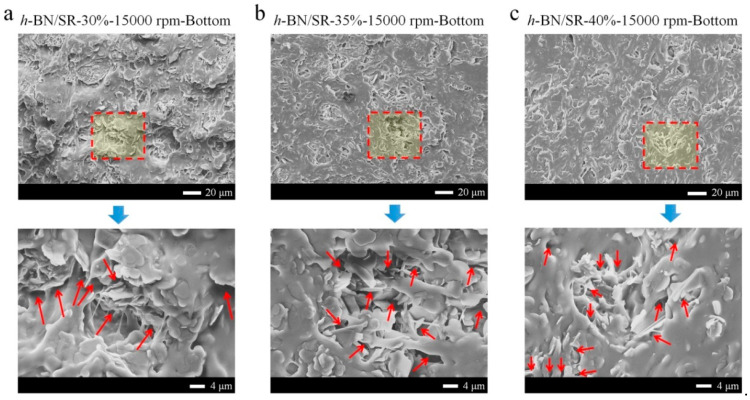
(**a**–**c**) SEM images of the cross-sections of *h-*BN/SR composites prepared with hypergravity treatments. Many micro-cracks (red arrows) can be seen between *h-*BN sheets.

**Figure 6:**
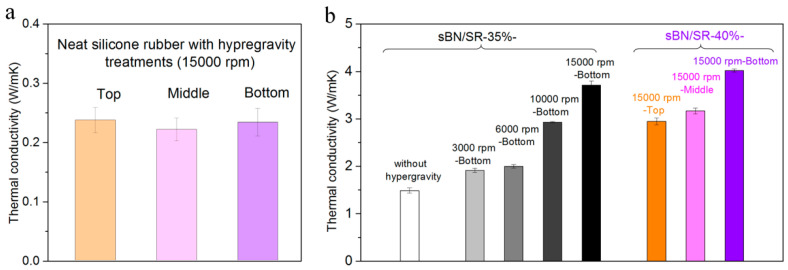
Comparison studies on the thermal conductivity of neat silicone rubber and BN/SR composites. (**a**) The through-plane thermal conductivity of neat silicone rubber prepared with hypergravity treatments. (**b**) The through-plane thermal conductivity of *h-*BN/SR and sBN/SR composites prepared with different methods.

**Figure 7:**
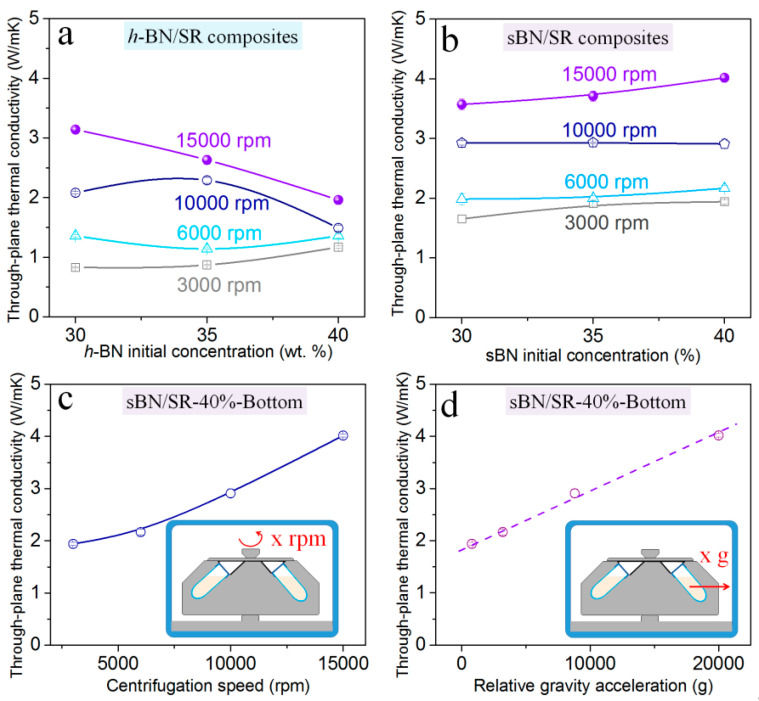
Factors affecting the thermal conductivity of BN/SR composites prepared with hypergravity treatments. (**a**) The effect of *h-*BN initial concentration on the thermal conductivity of *h-*BN/SR composites. (**b**) The effect of sBN initial concentration on the thermal conductivity of sBN/SR composites. (**c**) The effect of centrifugation speed and (**d**) relative acceleration on the thermal conductivity of sBN/SR-40%-bottom composites prepared with hypergravity treatments.

**Figure 8:**
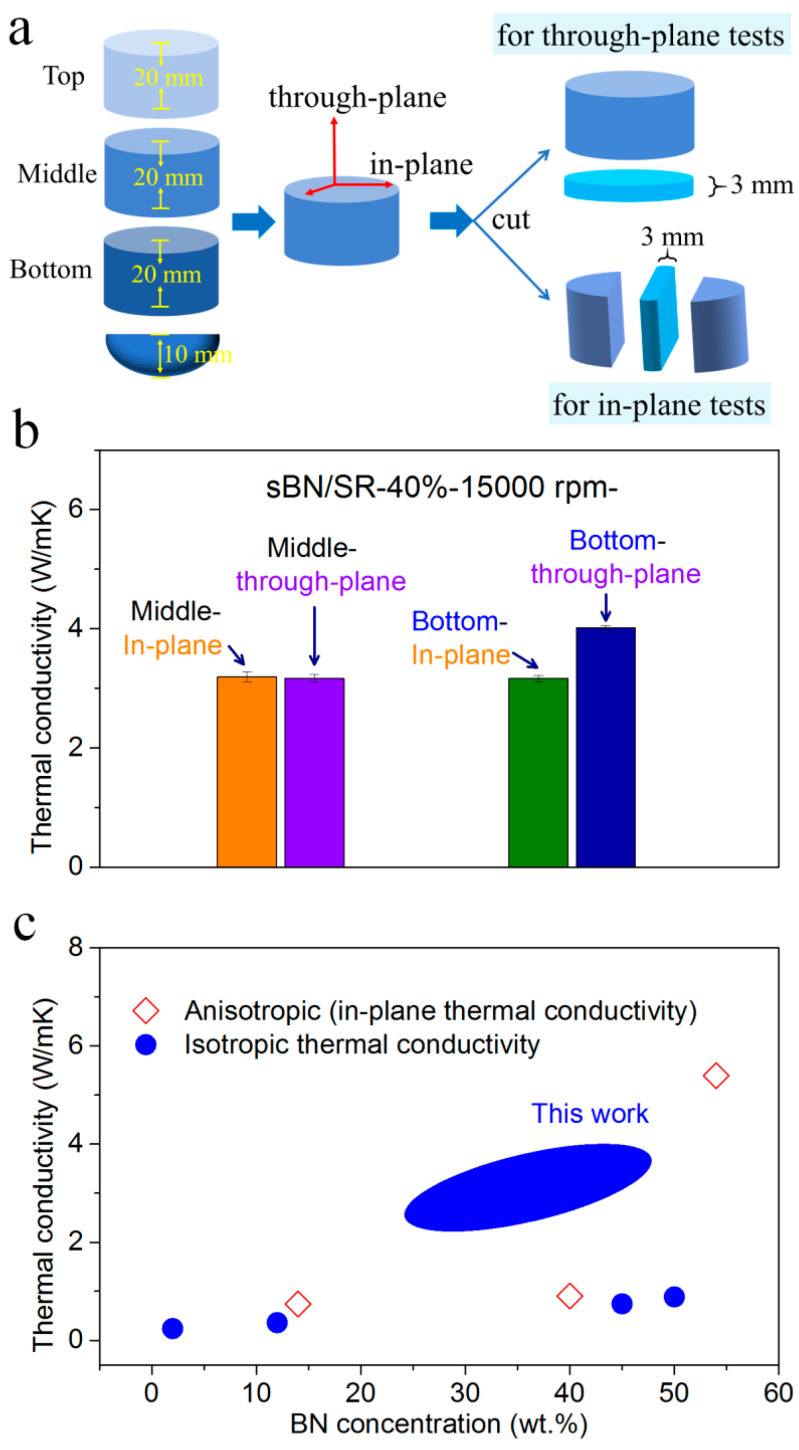
(**a**) Scheme diagram of the samples for through-plane and in-plane thermal conductivity tests. (**b**) The thermal conductivity in different parts and different directions of sBN/SR-40%-15,000 rpm composites. (**c**) Comparison between the thermal conductivity of BN/SR composites in the literature and in this work.

**Figure 9:**
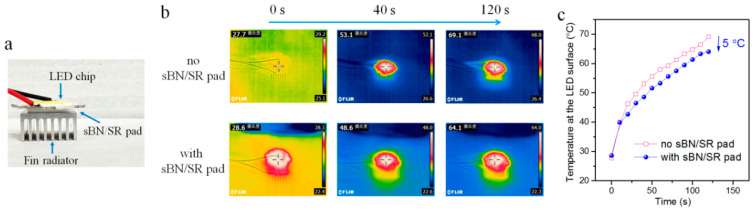
Demonstration of the thermal interface applications of sBN/SR composites. (**a**) Photograph showing the thermal interface application. (**b**) Thermal infrared images of the devices with or without sBN/SR composites. (**c**) The recorded maximum temperature on the light-emitting diode (LED) chip surface.

In this contribution, we report a new strategy to improve the thermal conductivity of polymer composites (filled type). This strategy is named a “hypergravity accumulation strategy” because its key step is treating the raw material mixtures of thermal conductive polymer composites in hypergravity fields before polymerization or cooling (Figure 1). The hypergravity treatments can force thermal conductive fillers (Figure 2) to settle and accumulate (Figure 3), resulting in compacted microstructures, increased thermal conductive pathways, and reduced defects in the obtained composites (Figure 4 and Figure 5). All these are beneficial to the thermal conductivity of polymer composites (Figure 6, Figure 7 and Figure 8). Moreover, this strategy is a universal method that applies to various fillers, polymers, or preparation techniques (Figure 1). Here we take boron nitride/silicone rubber (BN/SR) composites as an example to show the details of the hypergravity accumulation strategy. BN is chosen as the filler because: (i) it is one of the most promising thermal conductive fillers, owing to its high thermal conductivity (100–5000 W/mK), good electrical insulation properties, low density (~2.2 g/cm^3^), and economic affordability [34,35,36,37,38]; (ii) it is difficult to disperse BN in a polymeric matrix homogenously due to its low surface energy and poor wettability [39,40,41,42,43]; and (iii) BN-filled polymer composites usually have a thermal conductivity lower than 3 W/mK (Figures S1–S4), despite the high thermal conductivity of BN itself, and this is a quite strange and interesting problem worth study. Silicone rubber is chosen as the matrix material mainly because silicone rubber-based thermal conductive composites such as thermal conductive silicone pads, gap fillers, and thermal conductive silicone gels are very popular in practical applications. Comparison studies were made between two kinds of BN, including pristine *h-*BN and spherical assembled *h-*BN (sBN). Moreover, thermal interface performance of BN/SR composites were studied (Figure 9), and a systematic statistical study on the thermal conductivity of BN/polymer composites was made (Figures S1–S5). The results below will show a new, highly efficient, and simple strategy to improve the thermal conductivity of polymer composites.

## 2. Materials and Methods

### 2.1. Materials

*h-*BN (powders, average size 5 µm, purity 99%) and sBN (powders, average size 54 µm, purity 99%) were purchased from Shanghai Bestry Performance Materials Co., Ltd. (Shanghai, China). Dimethyl silicone oil (AR) was bought from Tianjin Chemat Chemical Technology Co., Ltd. (Shanghai, China). Silicone rubber precursors (addition-type mold silicone, A-B type, used as received) were purchased from Taida Chemical Raw Materials Wholesale on the website of Taobao.com (China). Poly(ethylene glycol) (PEG, average molecular weight 1000, AR) was purchased from Shanghai Yien Chemical Technology Co., Ltd. (Shanghai, China). Poly(vinyl alcohol) (PVA, type 1799, alcoholysis degree 98~99%, AR) were supplied by Shanghai Aladdin Biochemical Technology Co., Ltd. (Shanghai, China).

### 2.2. Preparation of BN/SR Composites with Hypergravity Treatments

BN/SR composites were prepared with hypergravity treatments by the in-situ polymerization method (Figure 1). Below we introduce detailed procedure of sBN/SR composites. *h-*BN/SR composites were prepared by the same procedure, and the only difference was replacing sBN with *h-*BN.

In the case of sBN/SR composites, sBN and silicone rubber raw materials (mass ratio of silicone Part A to silicone Part B and to dimethyl silicone oil: 10:1:11) were mixed in a beaker with mechanical stirring (2 min). The initial concentration of sBN in the mixtures was 30, 35, or 40% (mass percentage, wt.%), respectively. The mixtures were transferred into an agate mortar and ground for 30 min to obtain raw material mixtures. Hypergravity treatments were performed on a centrifuge (H2500R, Xiangtan Instruments Co., Ltd., Xiangtan, China). The raw material mixtures were transferred into centrifugal tubes (30 mL) and centrifuged for 30 min (can be other centrifugation time, if necessary). The centrifugal speed was set as 3000, 6000, 10,000, or 15,000 rpm, respectively (other centrifugation speeds can be used, if necessary). Then, the centrifugal tubes were heated in a blast oven (60 °C, 2 h; then 120 °C, 2 h) to cure the mixtures. The products were moved out from the centrifugal tubes and cut into specimens (about 3 mm thick, Figure 1 and Figure 3) with a sharp blade. The samples are named “sBN/SR-x%-y rpm-z”, where x is the concentration of sBN in the raw material mixtures, y is the centrifugation speed, and z (bottom, middle, or top) is the position where the specimens were cut (Figure 3c).

### 2.3. Preparation of sBN/SR Composites without Hypergravity Treatments

sBN powders were mixed with silicone rubber precursors (see Section 2.2) by mechanical stirring (2 min) and grinding (30 min). The mixture was degassed in a vacuum oven (20 min), molded in stainless steel casting mold, and heat-cured in an oven (60 °C, 2 h; then 120 °C, 2 h). The concentration of sBN in the composites was 35%. We tried higher concentrations (40%) but failed because the resultant raw material mixtures could not be mixed well by mechanical stirring.

### 2.4. Preparation of BN/PEG Composite with Hypergravity Treatments

BN/PEG composites were prepared by the melting blending method. The initial concentration of sBN was 35 wt.%. Solid PEG was heated and melted at 100 °C in an oven. sBN and liquid PEG were mixed in a beaker and stirred for 2 min, followed by 10 min of grinding in an agate mortar. In this process, the mixture was heated at 80 °C to maintain PEG in a molten state. Then the mixture was transferred into centrifugal tubes and centrifuged (15,000 rpm, 30 min). sBN/PEG composites were obtained after the mixture in centrifugal tubes was fully cooled to room temperature. The composite was cut into specimens (Figure 1) with a blade. This composite was prepared to prove the feasibility of hypergravity accumulation strategy in the melting blending techniques.

### 2.5. Preparation of BN/PVA Composite with Hypergravity Treatments

BN/PVA composites were prepared by the solvent blending method. PVA powders were mixed with deionized water in a beaker and stirred with a glass rod for 30 min at room temperature. The dispersion was heated for 1 h at 100 °C in a water bath pot (DF-101S, Gongyi Instrument Co., Ltd., Gongyi, China) and stirred using an electric blender (BOS-110, Shanghai Specimen and Model Factory, Shanghai, China) at 700 rpm to obtain a PVA aqueous solution with a concentration of 10 wt.%. PVA aqueous solution (56 g) and sBN (24 g) was mixed in a breaker with a stirring glass rod for 2 min. The obtained mixture was transferred into centrifugal tubes and centrifuged (15,000 rpm, 1 h). The obtained gel-like product was move out from the tubes and dried in an oven (50 °C, >24 h) to obtain sBN/PVA composites. This composite was prepared to prove the feasibility of hypergravity accumulation strategy in the solvent blending techniques.

### 2.6. Samples for Viscosity Tests

**Figure 10:**
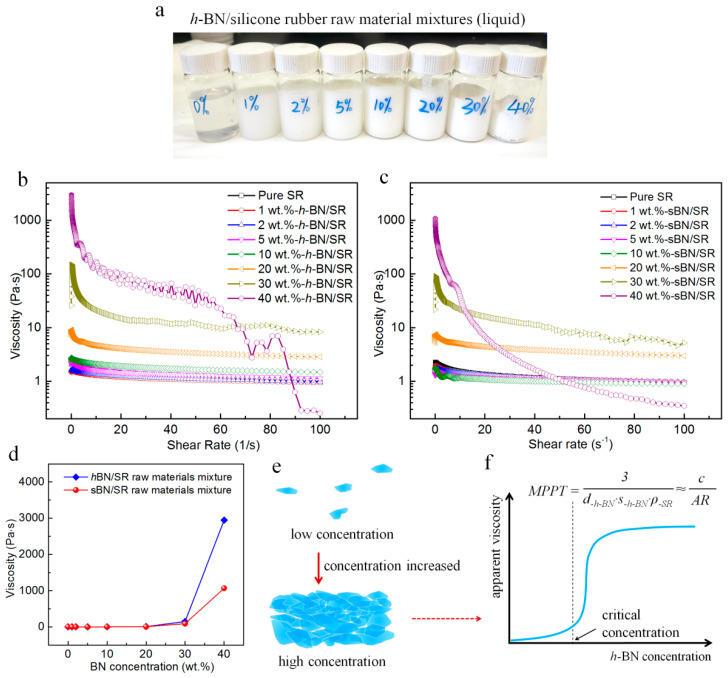
Studies on the high-viscosity problem. (**a**) Photograph showing some rheological test samples. (**b**) Shear viscosity curves of *h-*BN/SR raw material mixtures. (**c**) Shear viscosity curves of *s*BN/SR raw material mixtures. (**d**) Plots of the maximum viscosity on every curve against the BN concentration. (**e**) Scheme diagram of the dispersion states of *h-*BN sheets at low and high concentrations. (**f**) *h-*BN’s dispersion systems usually have a high-viscosity problem around their Most Probable Percolation Threshold (MPPT) concentration. It is mainly affected by the lateral size (*d*_-*h-*BN_) and specific surface area of *h-*BN (*S_-h-_*_BN_), and the density of silicone rubber’s raw materials (*ρ_-SR_*); or the aspect ratio of *h-*BN (AR) (c is an indeterminate coefficient).

The high-viscosity problem of BN/SR raw material mixtures was studied on a rotating rheometer. BN (*h-*BN or sBN) was mixed with silicone Part A and dimethyl silicone oil (silicone Part A:silicone oil mass ratio = 1:1) by the same mechanical mixing procedure of BN/SR composites. Every series of samples contained 8 kinds of mixtures with different BN concentrations (0, 1, 2, 5, 10, 20, 30, and 40 wt.%). A photo of some samples can be found in Figure 10a.

### 2.7. Characterization

Scanning electron microscopy (SEM) studies on BN powders and the cross-sections of BN/SR composites were conducted on a Zeiss Gemini SEM500 scanning electron microscope (Carl Zeiss, Oberkochen, Germany).

X-ray diffraction (XRD) of sBN and sBN/SR composites was obtained on an X-ray diffractometer (D8 advance, Bruker, Germany).

Thermal conductivity of BN/SR composites was measured on a DRL-III thermal conductivity meter (Xiangtan Instrument, Xiangtan, China) by the steady heat flow method according to ASTM D5470.

The density of different parts (bottom, middle, and top) of silicone rubber and BN/SR composites was measured with water, cylinders, and an electronic balance by the Archimedes’ method.

Thermal interface applications of sBN/SR composites were studied with a home-made set-up consisting of electrical source (FS4-7 W, ordered on the website of Taobao.com, China), a light-emitting diode (LED) chip (power 5 W, ordered on the website of Taobao.com, China), a copper fin radiator (20 mm × 20 mm, ordered on the website of Taobao.com, China), and a thermal infrared imager camera (FLIR E8 Wifi, FLIR Systems Inc., Wilsonville, OR, USA). A sBN/SR pad (15 mm × 15 mm × 1.5 mm) was put between the LED chip and copper fin radiator. In a test, the LED chip was turned on, and the temperature on the chip surface was recorded with the thermal infrared imager camera.

Shore C hardness of BN/SR composites was tested on a hardness tester (LX-C, Xijing Instrument and Chemicals Co., Ltd., Xi’an, China).

The shear viscosity of BN/SR raw material mixtures was measured on an MCR 302 rotating rheometer (Anton Paar GmbH, Graz, Austrian) with an increasing shear rate from 1 s^−1^ to 100 s^−1^.

## 3. Results

### 3.1. Hypergravity-Induced Accumulation of BN

The shape and size of fillers have great influence on the structures and properties of polymer composites (filled type). SEM profiles show that *h-*BN particles are 2D sheets (Figure 2a), and sBN particles are spherical particles with an average size of 54 ± 18 µm (Figure 2b,c). The higher multiple SEM image shows that sBN consists of thin sheets (Figure 2d). The XRD pattern of sBN (Figure 3f) is a typical pattern of *h-*BN (Figure 3d). Therefore, sBN is made of *h-*BN sheets in a spherical assembly manner.

The raw materials of BN/SR composites were treated in a hypergravity field before heat-curing. The main purpose of hypergravity treatments was to force the raw material to settle and accumulate in containers (Figure 3b). The hypergravity field was created on a centrifuge. The hypergravity, or the acceleration, was controlled by centrifugation speed. In this work, four typical centrifugation speeds were used for comparison studies (Table 1).

High acceleration may lead to accumulation or separation of some components in the raw material mixtures. This may affect the preparation and structures of BN/SR composites. This is a new problem that has not been studied before. Therefore, it is necessary to clarify the influence of hypergravity treatments. 

At first, neat silicone rubber was studied. The raw material mixture of neat silicone rubber after hypergravity treatments was successfully heat-cured. The resultant product was soft and semi-transparent (Figure 3a), just like normal silicone rubber. The density in different positions of the product was always about 1.0 g/cm^3^. These imply that hypergravity treatments did not cause obvious phase separation or accumulation in the raw material mixture of neat silicone rubber. This is probably because the raw material components, such as vinyl silicone oil, dimethyl silicone oil, and hydrogen containing silicone oil, have similar density.

Then we studied BN/SR composites prepared with hypergravity treatments. The composites are white and non-transparent (Figure 3a). It is supposed that BN particles tend to settle in containers when the raw material mixtures are treated in a hypergravity field because BN has higher density (~2.2 g/cm^3^) than silicone rubber (~1.0 g/cm^3^). Comparison studies were conducted between specimens from three parts of BN/SR composites: top, middle, and bottom (Figure 3c). Figure 3d shows the XRD patterns of *h-*BN and *h-*BN/SR composites. *h-*BN has a typical (002) peak at 2θ = 26.5°. The specimens cut from the bottom of BN/SR composites have stronger (002) peaks (Figure 3d,f) and higher density (Figure 3e) than the others. Therefore, there are more BN particles in the bottom of the composites. It confirms that hypergravity treatments caused accumulation of BN in the bottom part of the composites, as illustrated in Figure 3b.

### 3.2. The Microstructures of BN/SR Composites

In order to figure out the effect of hypergravity on the composites’ microstructures, three kinds of BN/SR composites were prepared for comparison, including: (i) sBN/SR composites prepared without hypergravity treatments (Figure 4a,b), sBN/SR composites prepared with hypergravity treatments (Figure 3a and Figure 4c,d), and *h-*BN/SR composites with hypergravity treatments (Figure 5).

Figure 4a shows a digital photo of a normal sBN/SR-35% composite prepared by mechanical mixing and molding, which was a conventional preparation route without hypergravity treatments (Figure 1). Figure 4b shows its cross-section observed by SEM. Many micro-cracks (red arrows) can be seen around the fillers (Figure 4b). Such cracks will increase phonon scattering and interface thermal resistance, leading to decreased thermal conductivity. Figure 4c shows the cross-section SEM images of the sBN/SR-30%-3000 rpm composite prepared with hypergravity treatments. The fillers are mostly homogenously dispersed. In the top position of the composites, there are still many cracks around the fillers. In the middle position, there are fewer cracks. In the bottom position, there are substantially fewer cracks. In the case of the sBN/SR-40%-15,000 rpm composite (Figure 4d), there are some cracks in the top position, very few cracks in the middle position, and almost no cracks in the bottom position. Based on these results, it is evident that hypergravity treatments can make the microstructures of sBN/SR composites more compacted.

The microstructures of *h-*BN/SR composites are shown in Figure 5. All the SEM images show that *h-*BN sheets have been uniformly dispersed, and there are always some micro-cracks even when the composites were treated at 15,000 rpm (Figure 4d). The micro-cracks seem to be very similar, although the composites have different initial concentrations of *h-*BN. Most of the micro-cracks are seen between *h-*BN sheets. We suppose they are formed due to a “contact state” of *h-*BN sheets. This will be discussed in the Discussion section.

### 3.3. The Thermal Conductivity of BN/SR Composites

Due to the accumulation effect, the bottom part of BN/SR composites has higher BN concentration (Figure 3) and more compacted microstructures (Figure 4) than the top or middle parts. Such un-uniform structures may bring un-uniform thermal conductivity to the composites. This is a new problem to be studied.

The thermal conductivity in different parts of neat silicone rubber is almost the same (~0.2 W/mK, Figure 6a), suggesting that hypergravity did not cause accumulation in neat silicone rubber. This agrees with the density results (Section 3.1).

BN/SR composites have higher thermal conductivity than neat silicone rubber, particularly when the composites were prepared with hypergravity treatments. For instance, sBN/SR-35% composite prepared without hypergravity treatments has a thermal conductivity of 1.5 W/mK, sBN/SR-35% composites prepared with hypergravity treatments have higher thermal conductivity (1.9–3.7 W/mK), and the sBN/SR-40%-15,000 rpm composite has the highest (4.0 W/mK) (Figure 6b). Therefore, hypergravity treatments can obviously improve the thermal conductivity of BN/SR composites. This should be attributed to the accumulation effect and the compacted microstructures caused by the hypergravity treatments.

There are many factors affecting the thermal conductivity of BN/SR composites. Figure 7 shows the influence of BN initial concentration and centrifugation speed. It should be noted that all the thermal conductivity in Figure 7 refers to the through-plane thermal conductivity in the bottom position of BN/SR composites (Figure 8a).

When the initial concentration of *h-*BN is fixed at 30 wt.%, the thermal conductivity is 0.8–3.2 W/mK (Figure 7a). In the case of sBN/SR composites (Figure 7b), the sBN/SR-30%-3000 rpm composite has a thermal conductivity of 1.6 W/mK, while the sBN/SR-30%-15,000 rpm composite has an obviously higher value (3.6 W/mK). Therefore, it can be concluded that the thermal conductivity of BN/SR composites increases with the centrifugation speed when the initial concentration of BN is fixed (Figure 7a–c). The thermal conductivity in the bottom of sBN/SR-40% composites is plotted against the centrifugation speed (Figure 7c) and acceleration (Figure 7d), respectively. It is interesting that the thermal conductivity in the bottom of sBN/SR-40% composites increases almost linearly with the acceleration (Figure 7d). These prove that high centrifugation speed (or high acceleration) can promote the increase in thermal conductivity. Moreover, it is visible that sBN/SR composites possess higher thermal conductivity than *h*-BN/SR composites when they have the same initial concentrations of BN (Figure 7a,b).

When the centrifugation speed is fixed, *h-*BN/SR composites and sBN/SR composites present different trends. As shown in Figure 7a, the thermal conductivity of *h-*BN/SR composites increases only a bit (at 3000 rpm or 6000 rpm) or even decreases (10,000 rpm or 15,000 rpm) with the initial concentration of *h-*BN. In contrast, the thermal conductivity of sBN/SR composites always increases with the initial concentration of sBN. This difference is related to the micro-cracks in the composites (Figure 5) and the high-viscosity problem, which will be discussed in Section 4.

From Figure 3, Figure 4, Figure 5, Figure 6 and Figure 7, it is clear that hypergravity treatments cause un-uniform structures and properties along the axial direction. It leads to a question that whether the thermal conductivity is different at the through-plane and in-plane directions of the composites. In order to clarify this, we produced through-plane and in-plane samples and tested their thermal conductivity for comparison (Figure 8a).

Figure 8b suggests that the sBN/SR-15,000 rpm composite has the same in-plane and through-plane thermal conductivity in the middle position (3.2 W/mK). It has higher thermal conductivity at the through-plane direction than at the in-plane direction in the bottom position. Therefore, both isotropic and quasi-isotropic thermal conductivity exist in sBN/SR composites. The quasi-isotropy means the thermal conductivity at two directions is quite close (in one direction 4.0 W/mK and in the other direction 3.2 W/mK), which is quite different from the conventional anisotropic thermal conductivity reported in the literature [44,45]. If researchers need BN/SR composites with high and completely isotropic thermal conductivity, they can prepare the composites with hypergravity treatments and then cut and collect a part of the composites. Although this cutting operation may waste some materials, the collected part has high and isotropic thermal conductivity which is difficult to obtain by the conventional methods, and, hence, it is acceptable in many cases.

Figure 8c shows a thermal conductivity comparison between the BN/SR composites in the open literature and in this work. As a kind of composite with isotropic thermal conductivity, our BN/SR composites prepared with hypergravity treatments have obviously higher thermal conductivity than those in the literature [46,47,48]. Our thermal conductivity is even higher than that of some BN/SR composites with anisotropic structures [49,50]. This is achieved without any surface modification of BN, 3D assembly of BN, or synergism between different fillers. Therefore, the hypergravity accumulation strategy is really efficient. Moreover, we conjecture that even higher thermal conductivity can be achieved if the hypergravity accumulation strategy is carried out in combination with some other strategies such as surface modification of BN or using synergistic fillers.

### 3.4. Possible Applications

Due to the high thermal conductivity, BN/SR composites have potential in cooling applications. Here we demonstrate a simple thermal interface application in an LED cooling system. Figure 9a shows the application. A sBN/SR-40%-15,000 rpm-Bottom pad (about 3 mm thick) was put between a heat sink (fin radiator) and a LED chip (power: 5 W). The LED chip was on, and the temperature on the chip surface was recorded using a thermal infrared imager camera (Figure 9b). Figure 9c shows that the maximum temperature on chip surface was decreased by about 5 °C when a sBN/SR pad was used. Therefore, BN/SR composites prepared with hypergravity treatments can be used to promote the heat spread in cooling systems. 

However, it should be noted that the practical cooling performance of sBN/BR pad, which is a 5 °C temperature decrease, is not good enough. It is worse than using normal 3.5 W/mK alumina/silicone rubber pads (10–20 °C temperature decrease; we have another on-going study on this topic) although it has higher thermal conductivity (4.0 W/mK). This is probably because the sBN/SR pad had much higher hardness (Shore C > 70) than the alumina/silicone rubber pad (Shore C 20–40). In thermal interface applications, a good contact between the surfaces of heat sink, heat source, and thermal conductive pad is very important. Hence, soft pads are preferred because they can form better contact with devices’ surfaces. Our sBN/SR pad is too hard. Its thermal interface application performance cannot be very good. This is a weak point of the BN/SR composites we reported here. In the future studies, it will be necessary to decrease the hardness of the composites.

## 4. Discussion

In the above sections, the preparation procedure, hypergravity-induced accumulation of BN, the microstructures and thermal conductivity of BN/SR composites, and thermal interface applications of sBN/SR composites are presented. There remain some small but interesting problems to be discussed. All these problems are related to a basic problem: the high-viscosity problem of BN/SR raw material mixtures.

### 4.1. The High-Viscosity Problem

The high-viscosity problem refers to a common phenomenon in that the raw material mixtures (liquid) of polymer composite, such as BN/SR raw materials mixtures, graphene/epoxy resin mixtures, and graphite/plastic molten mixtures, become too viscous to mix when the filler concentration is high enough.

Here we study the shear viscosity of BN/SR raw materials. Figure 10a shows a digital photograph of *h-*BN/SR raw material mixtures (test samples). Figure 10b,c show the shear viscosity curves of the raw material mixtures of *h-*BN/SR composites and sBN/SR composites, respectively. It is clear that the BN/SR mixtures became shear-thinning liquid. The maximum value on every curve is plotted against BN concentration (Figure 10d). It is noticeable that the viscosity values have a huge increase when BN concentration reaches 30 wt.%, and *h-*BN brings much more viscosity increase than sBN.

The huge viscosity increase can be explained by the Most Probable Percolation Threshold (MPPT) theory we developed recently (Figure 10e,f) [51,52]. When the *h-*BN concentration in *h-*BN/SR raw material mixtures is small, *h-*BN sheets are mostly isolated sheets that do not touch neighboring ones (Figure 10e). When the *h-*BN concentration reaches a critical concentration, *h-*BN sheets begin to touch neighboring ones and form a jamming effect when the concentration is high enough, resulting in huge increase in the apparent viscosity of raw material mixtures. This huge increase is mainly due to the jamming effect among *h-*BN sheets, and it is mainly affected by the aspect ratio (or the lateral size and specific surface area) of *h-*BN sheets (seeing the MPPT equation in Figure 10f). *h-*BN has a larger aspect ratio than sBN, and hence leads to more viscosity increase. Other 2D materials such as graphene, graphite nanoplatelets, MoS_2_, layered clay have similar sudden viscosity increase phenomena in their dispersion systems (solutions, colloids, suspensions, filled type composites, etc.) [51].

### 4.2. The Micro-Cracks in BN/SR Composites

The micro-cracks seen in Figure 4 (sBN/SR composites) and Figure 5 (*h-*BN/SR composites) seem to be two different cases. The micro-cracks in sBN/SR composites are seen between sBN particles and silicone rubber (Figure 4). They are formed mainly because BN has low surface energy and poor wetting with the matrix. Surface modification of BN may help to improve the wetting and dispersibility and remove such micro-cracks.

There are two types of micro-cracks in *h-*BN/SR composites. One is formed between *h-*BN sheets and silicone rubber, just like those in sBN/SR composites. This type is also attributed to the low surface energy and poor wetting of *h-*BN. The other is formed between *h-*BN sheets, and this is the main type. This type of micro-crack is formed mainly due to the 2D layered structure of *h-*BN sheets and the high-viscosity problem of *h-*BN/SR raw material mixtures. As illustrated in Figure 10e, when the concentration of *h*-BN is high enough (>25%, according to Figure 10d), *h-*BN sheets become “touched sheets” which means the sheets touch neighboring ones and this leads to a jamming effect. *h-*BN sheets support their neighboring ones and hence create small spaces (like holes) between them. Due to the high viscosity, *h-*BN/SR raw material mixtures could not completely fill up these holes, resulting in many micro-cracks between *h-*BN sheets, as can be seen in Figure 4. Therefore, the micro-cracks in *h-*BN/SR composites should be mainly attributed to the high 2D structures of *h*-BN and the viscosity problem *h-*BN/SR raw material mixtures.

### 4.3. The Thermal Conductivity of BN/SR Composites in the Literature and in This Work

In recent years, 2D highly thermal conductive materials such as graphene and *h-*BN have gained great research interests. These 2D materials have much higher thermal conductivity (200–5000 W/mK) than the conventional thermal conductive fillers such as alumina (20–40 W/mK). However, polymer composites filled with 2D materials usually have lower thermal conductivity than alumina-filled polymer composites. For instance, most of the graphene-filled polymer composites have a thermal conductivity lower than 5 W/mK [53,54,55]. Our statistical study shows that 67% of boron nitride (BN)/polymer thermal conductive composites reported in the open literature have a thermal conductivity lower than 3 W/mK (Figures S1 and S4), and most of the BN filled silicone rubber composites with an isotropic structure have a thermal conductivity lower than 1.0 W/mK (Figure 8c and Figure S6). In contrast, alumina-filled silicone rubber composites can reach a high thermal conductivity of 8–10 W/mK (no academic reference; many companies, including the corresponding author’s, can provide such products).

Therefore, here we see a very strange and interesting problem: for thermal conductive polymer composite (filled type) applications, 2D materials with extremely high thermal conductivity are not as good as spherical fillers which have much lower thermal conductivity. This mismatch can be mainly attributed to the serious high-viscosity problem in the preparation of 2D materials/polymer composites (filled type).

The thermal conductivity of polymer composites is largely affected by the concentration of thermal conductive fillers. When the filler concentration is small, the thermal conductivity usually increases slowly with the concentration. Once the concentration reaches a critical value, the thermal conductivity may increase rapidly with filler concentration. As a result, high filler concentration is usually necessary in the preparation of highly thermal conductive polymer composites.

However, as we have discussed above, high filler concentration may result in high apparent viscosity of raw material mixtures. Due to the high-viscosity, the raw material mixtures of 2D fillers/polymer composites have poor flowability, and are thus difficult to mix and process. Sometimes, the flowability is so poor that the raw material mixture cannot fill up the small holes between 2D fillers, resulting in micro-cracks in the composites, as can be seen in Figure 5. Meanwhile, due to the high viscosity, it is hard to increase the 2D filler concentration to reach a high level. For instance, most of the graphene-filled polymer composites or *h-*BN-filled polymer composites have a filler concentration lower than 50 wt.%, while spherical alumina-filled silicone rubber can have a filler concentration as high as 96% (no academic reference; many companies, including the corresponding authors’, can provide such products).

In one word, the high-viscosity usually brings decreased flowability of raw material mixtures, increased micro-cracks in composites, and limited filler concentration. All these are against the increase in thermal conductivity in 2D materials-filled composites. This is why the graphene-filled polymer composites and *h-*BN-filled polymer composites reported in the open literature mostly have quite low thermal conductivity, although graphene and *h-*BN themselves have extremely high thermal conductivity. Moreover, Figure 10 implies that spherical fillers lead to a weaker high-viscosity problem than 2D fillers. This is why spherical fillers are more popular in the industrial manufacturing of thermal conductive polymer composites.

From these results and discussions, we feel that it is necessary to overcome or work round the high-viscosity problem when researchers want to develop highly thermal conductive 2D materials/polymer composites.

### 4.4. Advantages and Disadvantages of the Hypergravity Accumulation Strategy

In order to overcome the high viscosity problem, there are several possible strategies. The conventional 10 strategies to improve the thermal conductivity of BN/polymer composites are introduced in the Introduction section. Not all the 10 strategies can be used to overcome the high viscosity problem. The feasible strategies in these 10 strategies include the 3D assembly strategy [28,29,30] and the multi-recycles hot-pressing strategy [56], etc. However, from thee statistical study (see the Supplementary Materials), we find that the surface modification of BN, which has been regarded as one of the most promising strategies to improve the dispersibility and properties of BN/polymer composites, actually do not have obvious positive effects on the thermal conductivity of BN/polymer composites (Figure S3).

Another feasible strategy is making graphene or *h-*BN into spherical particles, because this will partially weaken the high-viscosity problem caused by the 2D structure of graphene or *h-*BN (Figure 10). However, as we can see in the previous research [57] and in Figure 10d, spherical fillers still lead to quite high viscosity in raw material mixtures. Therefore, the spherical strategy cannot solve the problem fundamentally.

In contrast, the hypergravity accumulation strategy we report here provides a new and better choice. The key idea of this strategy is using a hypergravity field, which is strong enough (>800 g) to overcome the high viscosity of raw material mixtures. The obtained composite has increased filler concentration and more compacted microstructures due to the accumulation effect. This is an advantage not available in the conventional methods. Moreover, this strategy is quite simple to operate because it only requires a high-speed centrifuge. Besides, the hypergravity accumulation strategy can be applied in different polymer composite systems. As shown in Figure 1, it can be used in three typical preparation techniques of polymer composites: in-situ polymerization (BN/SR composites), melting blending (BN/PEG composites), and solvent blending (BN/PVA composites). This indicates that the hypergravity accumulation strategy is a nearly universal strategy.

However, the hypergravity accumulation strategy also has disadvantages. For instance, its production scale is limited by the size of the centrifuge, and the products are usually not soft enough. Further efforts are necessary to improve this strategy. We believe that a good combination of an improved hypergravity accumulation strategy and the conventional strategies will lead to new and promising routes towards highly thermal conductive polymer composites. 

## 5. Conclusions

In this work, a hypergravity accumulation strategy has been developed to improve the thermal conductivity of polymer composites. The raw material mixtures of BN/silicone rubber composites were treated in hypergravity fields (800–20,000 g) before heat-curing. SEM studies proved that hypergravity treatments could make the microstructure of BN/SR composites much more compacted. The resultant composites had obviously improved thermal conductivity. The highest reached 4.0 W/mK. The improved thermal conductivity is mainly attributed to the accumulation effect of BN and the compacted microstructures in the composites, which were both caused by the hypergravity treatments. It was found that sBN was better than *h-*BN, and this is mainly because sBN has less of a high-viscosity problem. Thermal interface application study showed that the composites could help to decrease the temperature on LED chip by 5 °C. The mechanism of the improvements in the structures and properties of BN/SR composites, the high viscosity problem, and the advantages and disadvantages of the hypergravity strategy were discussed. We hope it will inspire research and applications of BN/polymer thermal conductive composites and other polymer composites.

## Figures and Tables

**Table 1:** Centrifugation speed and the corresponding relative gravity acceleration.

Centrifugation Speed (rpm)	Relative Gravity Acceleration (g)
3000	~800
6000	~3200
10,000	~8800
15,000	~20,000

## Data Availability

The data presented in this study are available on request from the corresponding author.

## Supplementary Materials

The following are available online at https://www.mdpi.com/2073-4360/13/3/459/s1, Figures S1–S6, Table S1, References.
