# Supplementary material for: Hypergravity-Induced Accumulation: A New, Efficient, and Simple Strategy to Improve the Thermal Conductivity of Boron Nitride Filled Polymer Composites
Source: Polymers (Basel). 2021 Jan 31;13(3):459. doi: 10.3390/polym13030459 (PMC7866976; doi:10.3390/polym13030459)
Supplement: Supplementary file 1 [file polymers-13-00459-s001.pdf]

Supplementary Materials of:

Hypergravity-induced accumulation: a new, efficient, and simple strategy to improve the thermal conductivity of boron nitride filled polymer composites

Kangkang Yu[a], Tao Yuan[a], Songdi Zhang[a], and Chenlu Bao[a,b,]*

[a]School of Materials Science and Engineering, Tiangong University, 399 Binshui West Road, Tianjin, 300387, China

[b]Tianjin HaiTe Thermal Management Technology Co., Ltd.. 6 Huake 8 Road, Tianjin, 300450, China

*Corresponding Authors. *E*-mail: bao_chenlu@qq.com (CL Bao)

This file includes:

1. Supplementary text

2. Supplementary Figure s1–s6

3. Table s1

4. Supplementary References

1. Supplementary text:

1.1. Data collected from the literature of BN/polymer thermal conductive composites.

In order to have an overview in this field, we collected the data in the literature (Table s1) and draw statistic diagrams (Figure s1-6).

1.2 The concentration where the highest thermal conductivity was obtained

Some literatures used volume percentage (vol.%), while some used weight percentage (wt.%). In order to make parallel comparison, all the volume percentage was transferred into weight percentage according to Eq. s1:

$$\text{wt.\%} = \frac{\text{vol.\%}\cdot 2.2}{\text{vol.\%}\cdot 2.2+(1-\text{vol.\%})\cdot \rho_{-\text{matrix}}} * 100\% \qquad \text{s1}$$

where $\rho_{\text{-matrix}}$ is the matrix materials' relative density to water.

Supplementary Figures

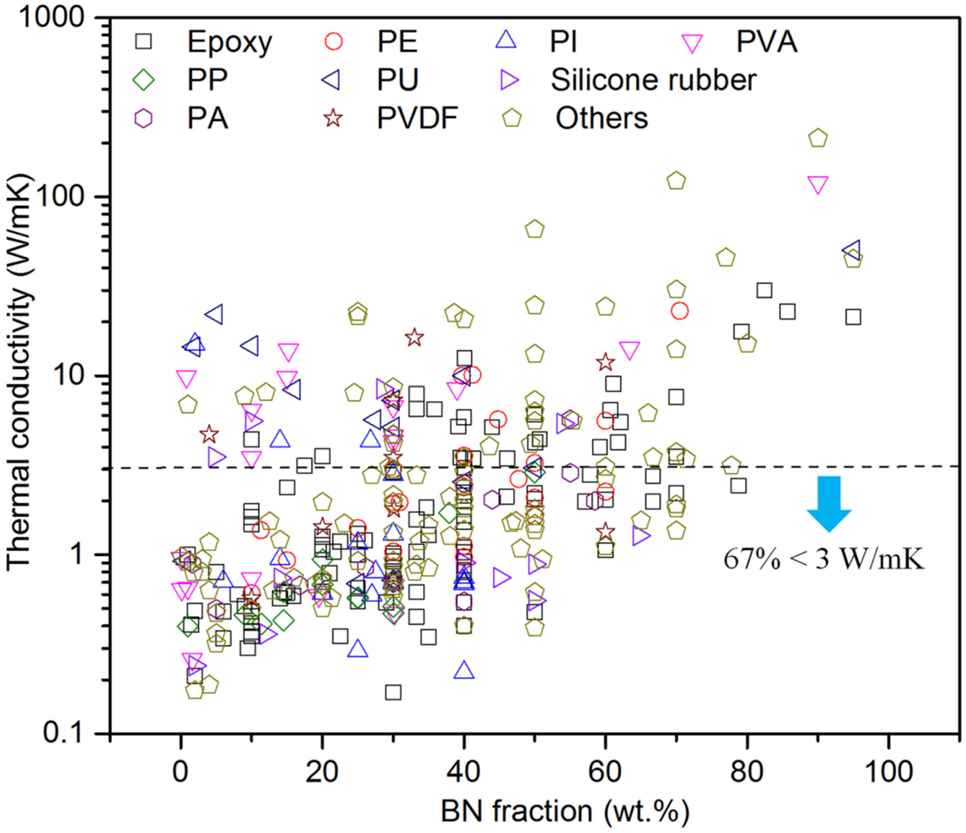

Figure s1. Parameter distribution diagrams colored according to polymer matrix.

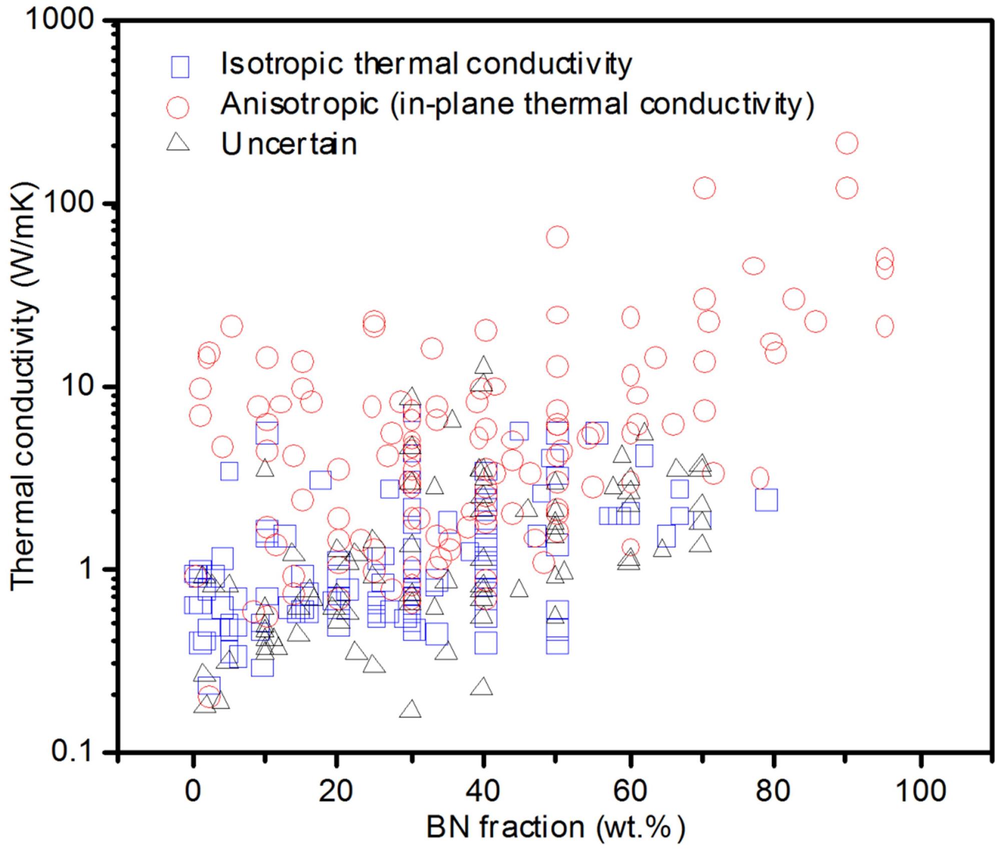

Figure s2. Parameter distribution diagrams colored according to the isotropy or anisotropy of thermal conductivity.

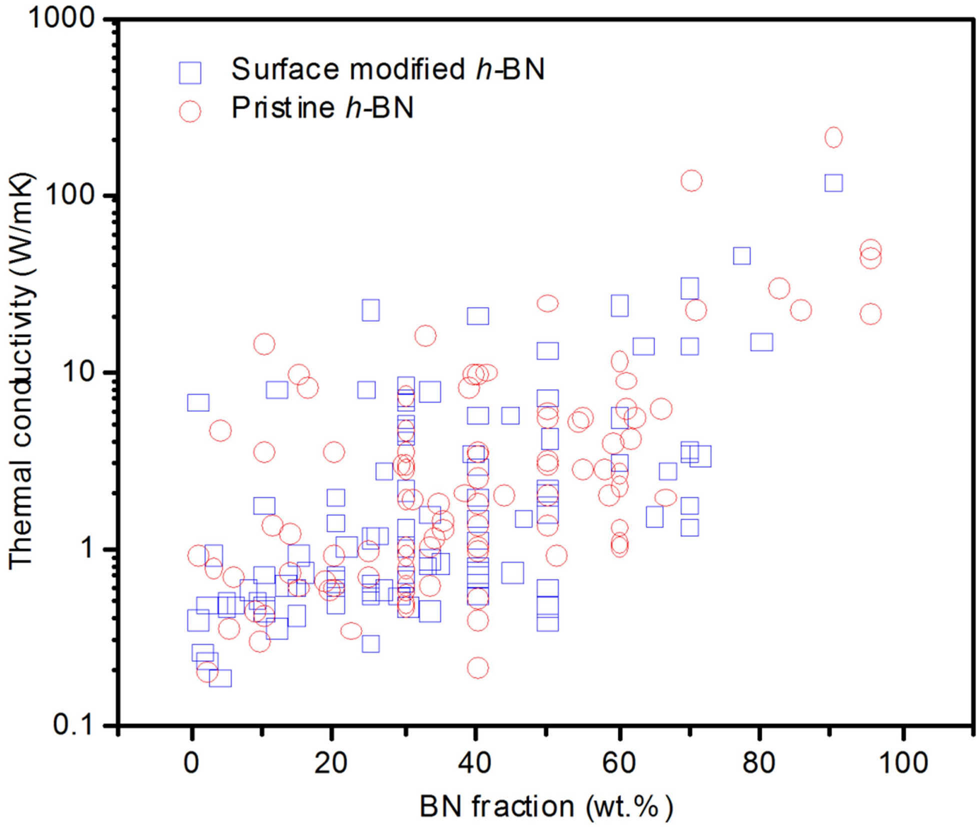

Figure s3. Parameter distribution diagrams colored according to the surface modification of *h*-BN. It implies that the surface modification of *h*-BN has little effect in improving the thermal conductivity of *h*-BN/polymer composites.

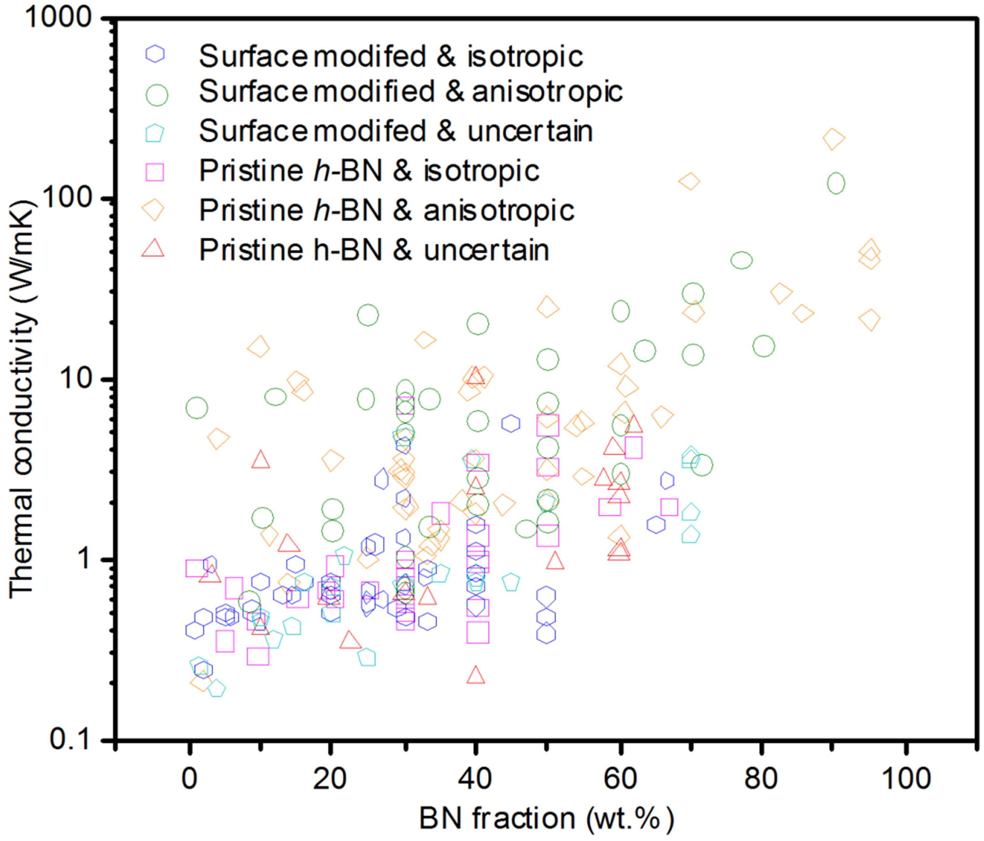

Figure s4. Parameter distribution diagrams colored according to both the surface modification of *h*-BN and the isotropy or anisotropy of thermal conductivity.

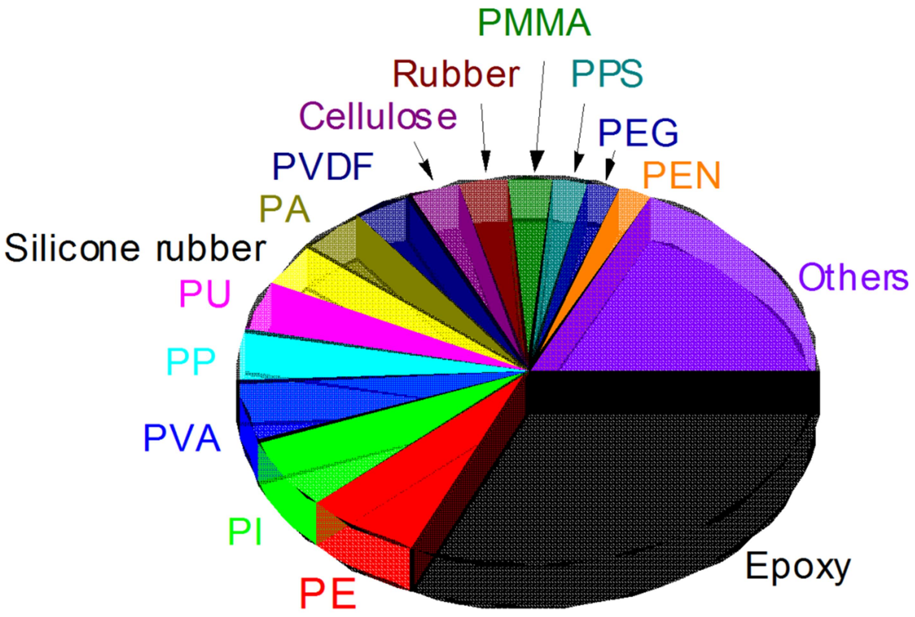

Figure s5. Pie chart of the most widely studied polymer matrix in BN/polymer thermal conductive composites. Abbreviations of polymers: polyethylene (PE), polyimide (PI), poly(vinyl alcohol) (PVA), polypropylene (PP), polyurethane (PU), polyamide (PA), poly (vinylidene fluoride) (PVDF), polymethyl methacrylate (PMMA), polyethylene glycol (PEG), polyphenylene sulfide (PPS), poly(arylene ether nitrile) (PEN).

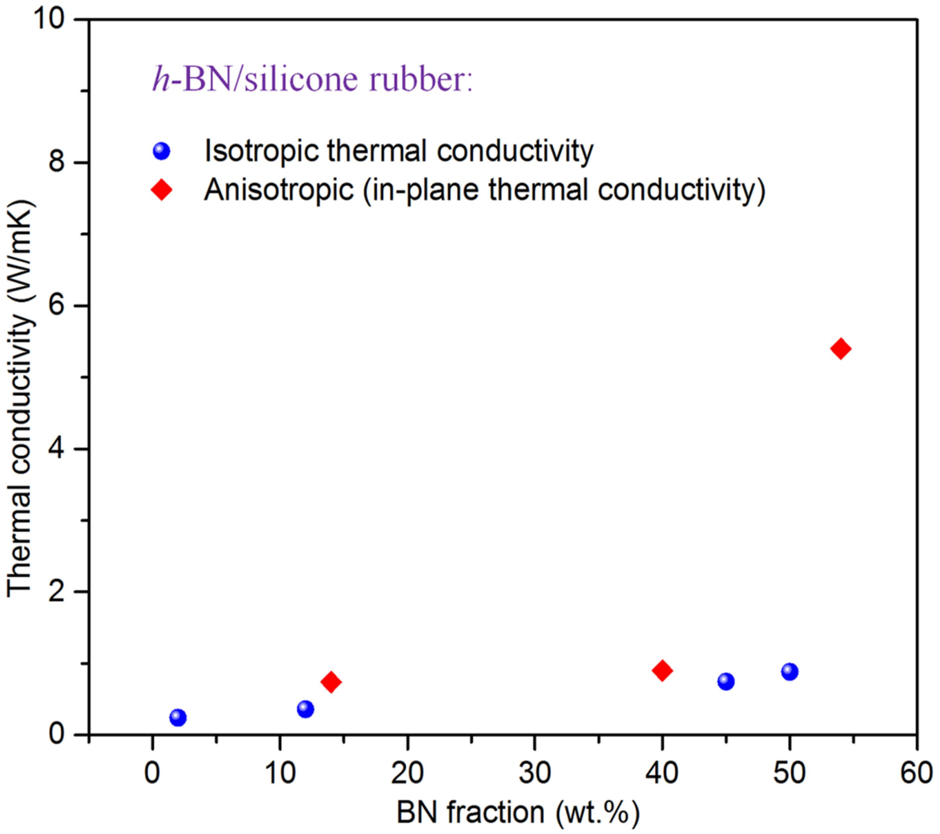

Figure s6. Parameter distribution diagrams colored according to isotropy or anisotropy of the thermal conductivity of *h*-BN/silicone rubber composites (without synergism fillers).

3. Table s1

Table s1 The data of BN/polymer thermal conductive composites reported in the literature

Citation	Matrix	Type of BN	Concentration of peak thermal conductivity	Highest thermal conductivity (W/mK)	Surface modified?	Anisotropic?
[1]	Epoxy	aluminum oxide and boron nitride	30 wt.%	0.17		
[2]	Epoxy	*h*-BN	2wt.%	0.21		yes
[3]	Epoxy	*h*-BN	5vol.%	0.3		
[4]	Epoxy	BN@PDA@Fe	6wt.%	0.34	yes	
[5]	Epoxy	BN	35 wt.%	0.3464		
[6]	Epoxy	*h*-BN	22.5 wt.%	0.35		not sure
[7]	Epoxy	surface-silanized BN	10 wt.%	0.35		
[8]	Epoxy	Functionalized Fe3O4/BNNs	10%	0.37		
[9]	Epoxy	*h*-BN	40%	0.4		
[10]	Epoxy	BNNT	1.5wt.%	0.407		
[11]	Epoxy	BN	10wt.%	0.415		
[12]	Epoxy	Functionalized h-BN	20vol.%	0.45	yes	
[13]	Epoxy	*h*-BN	10 wt.%	0.452	yes	
[14]	Epoxy resin -impregnated insulation paper	Na -*h*-BN	50wt.%	0.478	yes	
[15]	Epoxy	boron nitride (BN) modified by octadecyl trimethyl ammonium bromide	10%	0.48	yes	
[16]	Epoxy	*h*-BN	6%	0.48	yes	
[17]	Epoxy	*h*-BN	2%	0.484	yes	
[18]	Epoxy	*h*-BN	8.99%	0.517	yes	
[19]	Epoxy	*h*-BN	29 %	0.54	yes	
[20]	Ester/Epoxy	*h*-BN	40wt.%	0.54		
[21]	Epoxy	*h*-BN	25%	0.55	yes	
[22]	Epoxy	AlN/BN	42wt.%(2:1)	0.57		
[23]	Epoxy	BN-GS(graphene sponge)	15.9wt.%	0.588		
[24]	Epoxy	*h*-BN	8 wt.%	0.6	yes	yes
[25]	Epoxy	*h*-BN	15%	0.61		
[26]	Epoxy	*h*-BN	20 vol.%	0.618		
[27]	Epoxy	*h*-BN	20%	0.6188	yes	
[28]	Epoxy	*h*-BN	14.6%	0.62	yes	
[29]	Epoxy	*h*-BN	25wt.%	0.65	yes	
[30]	Epoxy	*h*-BN	20 wt.%	0.708	yes	
[31]	Epoxy	*h*-BN	20 wt.%	0.708	yes	

[32]	Epoxy	3D BN	25vol.%	0.72		yes
[33]	Epoxy	*h*-BN	30wt.%	0.78		
[34]	Epoxy	*h*-BN, *c*-BN	21%	0.788	yes	
[35]	Epoxy/polybutadiene	*h*-BN	40wt.%	0.8	yes	
[36]	Epoxy	SCF/Modified GNP-BN	3 wt.% SCF + 5 wt.% modified GNP-BN	0.8	yes	
[37]	Epoxy	*h*-BN	40%	0.82	yes	
[38]	Epoxy	BN	30%	0.92		
[39]	Epoxy	*h*-BN	40%	0.97		
[40]	Epoxy	*h*-BN	30wt.%	0.98		
[41]	Epoxy	*h*-BN	25wt.%	1		yes
[42]	Epoxy	MoS_2/*h*-BN	1%	1	yes	
[43]	Epoxy	*h*-BN	30 wt.%	1.02		
[44]	Epoxy	BN micosphere	40wt.%	1.03		
[45]	Epoxy	*h*-BN	12.1 vol.%	1.04	yes	not sure
[46]	Epoxy	*h*-BN	20 vol.%	1.04		yes
[47]	Thiol-Epoxy elastomers	micron boron nitride (mBN)	60 wt.%	1.058		not sure
[48]	Epoxy	*h*-BN	20 wt.%	1.07	yes	yes
[49]	Epoxy	BN and CNTs	40wt.%BN and 1wt.% of o-CNTs	1.1		not sure
[50]	Epoxy	*h*-BN@AgNPs	20wt.%	1.13		
[51]	Epoxy	dopamine modified micro-BN and KH550 modified na -Al2O3	22.5wt.% BN and 7.5 wt.% Al2O3	1.182	yes	not sure
[52]	Epoxy	*h*-BN	15vol.%	1.198	yes	
[53]	Epoxy	oCNTs@fBN	20 wt.%	1.26	yes	not sure
[54]	Epoxy	*h*-BN	35wt.%	1.29		yes
[55]	Epoxy	*h*-BN@PDA	25wt.%	1.31	yes	yes
[56]	Epoxy	*h*-BN	5.3vol.%	1.479		
[57]	Epoxy	Modified *h*-BN	40%	1.52	yes	
[58]	Epoxy	BN	20vol.%	1.577	yes	yes
[59]	Epoxy	BN na tube (BNNT)-	10wt.%	1.62	yes	
[60]	Epoxy	Boron Nitride Sphere (BNS)	40wt.%	1.66		
[61]	Epoxy	BNNP	10wt.%	1.75	yes	yes
[62]	Epoxy	*h*-BN	21vol.%	1.83		
[63]	Epoxy	BN/PS 3D skeletons	40vol.%	1.98	yes	
[64]	Epoxy	*h*-BN	50vol.%	1.98		
[65]	Epoxy	*h*-BN and graphite	60 wt.%	2.025		
[66]	Epoxy	*h*-BN	50%	2.05	yes	not sure
[67]	Epoxy	Benzyl alcohol modifiedBN (B-BN)	46 wt.%	2.11	yes	not sure
[68]	Epoxy	AgNPs and *h*-BNs	25 vol.%	2.14	yes	not sure
[69]	Epoxy	BN particles	70 wt.%	2.2		not sure
[70]	Epoxy	*h*-BN	50 wt.%	2.2	yes	yes

[71]	Epoxy	*h*-BN@GO	40wt.%	2.23	yes	
[72]	Epoxy	Spherical BN	15wt.%	2.37		yes
[73]	Epoxy	Al_2O_3@*h*-BN	65vol.%	2.43	yes	
[74]	Epoxy	*h*-BN	50vol.%	2.75	yes	
[75]	Epoxy-thiol system	*h*-BN	40 vol %	2.8		not sure
[76]	Epoxy	BNNT	30wt.%	2.9		not sure
[77]	Epoxy	*h*-BN	25.1 vol %	3.06	yes	not sure
[78]	Epoxy	*h*-BN	9.6vol.%	3.13		
[79]	Epoxy	*h*-BN	30 vol.%	3.445	yes	yes
[80]	Epoxy	*h*-BN@RGO	40wt.%	3.45	yes	not sure
[81]	Epoxy	*h*-BN–RGO	26.04 vol.%	3.45	yes	yes
[82]	Epoxy	BN	39.5wt.%	3.49	yes	not sure
[83]	Epoxy	BN	70 wt.%	3.521	yes	not sure
[84]	Glass fibers cloth/ Epoxy	spherical BN	20 wt.%	3.55		yes
[85]	Epoxy-thiol system	*h*-BN/spherical BN	42 vol %	4.0		not sure
[86]	Epoxy	BN	34.2 vol.%	4.24	yes	yes
[87]	Epoxy	*h*-BN	44.7vol.%	4.25		
[88]	Epoxy	*h*-BN	5.3 vol %	4.4		yes
[89]	Epoxy	Network of BN	34vol.%	4.42	yes	yes
[90]	Epoxy	Cu/h-BN	43.95wt.%	5.15		yes
[91]	Epoxy	hierarchical porous boron nitride (BN)	24.4 wt %	5.19		yes
[92]	Epoxy	*h*-BN	45vol.%	5.5		not sure
[93]	Epoxy	*h*-BN	40wt.%	5.86	yes	yes
[94]	Epoxy	oriented BN	50wt.%	6.09		yes
[95]	Epoxy	*h*-BN	43.6vol.%	6.418		yes
[96]	Epoxy	graphene and boron nitride	21.8/21.8 vol.%	6.5		not sure
[97]	Epoxy	*h*-BN	20vol.%	6.54		yes
[98]	Epoxy + PPS	spherical BN	70%	7.6	yes	yes
[99]	Mesophase pitch -based carbon fibers /Epoxy	*h*-BN	20vol.%	7.9	yes	yes
[100]	Epoxy	*h*-BN	44 vol.%	9		yes
[101]	liquid crystalline epoxy resin (LCER)	*h*-BN	25.1 vol.%	12.55	yes	not sure
[102]	Epoxy	hollow boron nitride microbeads (BNMB)	65.6 vol.%	17.61	可能	yes
[103]	Epoxy	*h*-BN	95wt.%	21.3		yes
[104]	Epoxy	*h*-BN	75vol.%	22.87		yes
[105]	Epoxy	*h*-BN	82.45wt.%	30.04		yes
[106]	polyethylene (PE)	*h*-BN	18.3vol.%	1.96		yes
[107]	PE	*h*-BN	50wt.%	3.25		
[108]	PE	*h*-*h*-BN	5.97 vol.%	1.37		yes
[109]	Low-density polyethylene (LDPE)	*h*-BN	25vol.%	1.12	yes	
[110]	LDPE	*h*-BN	27.63vol.%	2.65		
[111]	Hig*h*-density polyethylene	SC(stearyl	10wt.%	0.61	yes	not sure

	(HDPE)	chloride)-*h*-BN				
[112]	HDPE	*h*-BN	15%	0.93	yes	
[113]	HDPE	*h*-BN	50wt.%	2.08		yes
[114]	Linear-ultrahigh molecular weight polyethylene (LUHMWPE)	*h*-BNs	50 vol.%	23.03		yes
[115]	Ultrahigh molecular weight polyethylene (UHMWPE)	*h*-BN	21.6 vol %.	9.99		yes
[116]	HDPE	*h*-BN	25vol.%/60wt %	5.6	yes	yes
[117]	HDPE	*h*-BN	23.2vol.%	10.1		yes
[118]	Ultra high molecular weight polyethylene	BN	40wt.%	2.38		
[119]	HDPE	BN	40 wt %	3.57		yes
[120]	Ultra high molecular weight polyethylene	BN	25.93 vol.%	5.7	yes	
[121]	ultra-hig*h*-molecular-weigh t polyethylene	BNs/MWCNT	50 wt.%	1.641		not sure
[122]	HDPE	BN	60 wt.%	2.25		not sure
[123]	PE	micro-boron nitride (BN) and micro/na hybrid-BN particles	40%	0.97		
[124]	PTFE	*h*-BN	30vol.%	3		yes
[125]	Polytetrafluoroethylene (PTFE)	*h*-BN	30 vol.%	1.04	yes	yes
[126]	PTFE	*h*-BN	30vol.%	1.92		yes
[127]	PTFE	BNNs–GNs	25 wt.%	1.41		not sure
[128]	Polyimide (PI)	Micrometer BN/PAA	30wt.%	0.696	yes	
[129]	PI	CNF/h-BN	14.1vol.%	0.8	yes	yes
[130]	PI	BN	30wt.%	0.71	yes	yes
[131]	PI	*h*-BN	30wt.%	1.308	yes	
[132]	PI	BN-Fe-CNT	2wt.%	15	yes	yes
[133]	PI	*h*-BN	30wt.%	2.81		yes
[134]	PI	*h*-BNs	7 wt.%	2.95		yes
[135]	PI	AgNWs@BN	20 vol.%	4.33	yes	yes
[136]	PI	*h*-BN	40wt.%	0.22		
[137]	PI	Na -sized BN	25wt.%	1.16	yes	
[138]	PI	BN	20%	0.61		
[139]	PI	*h*-BN	40 wt.%	0.748	yes	
[140]	PI	*h*-BN	0.25 wt.%	0.291	yes	
[141]	PI	(CuNPs-CuNWs)@BN	10 wt %	4.32	yes	yes
[142]	PI	*h*-BN	40 wt.%	0.686		
[143]	PI	*h*-BN	40%	0.7032	yes	
[144]	PI	*h*-BN	27%	0.596	yes	
[145]	PI	*h*-BN	6%	0.711		
[146]	PI	BN/AlN	10 vol.%	0.95	yes	yes
[147]	Fluorinated PI	*h*-BN	14.2 vol.%	0.59		
[148]	poly(vinyl alcohol) (PVA)	*h*-BN	10wt.%	6.43		yes
[149]	PVA	OH-*h*-BN	0.12wt.%	0.64	yes	
[150]	PVA	BN@PEI	50vol.%	14.22	yes	yes

[151]	Polyacrylic acid (PAA) /PVA	BNNT	1wt.%	0.65	yes	
[152]	Polyethylhexylacrylate\Polyvinyl Alcohol(PEHA/PVA)	*h*-BN	10wt.%	improved by approximately 20 times		not sure
[153]	PVA	*h*-BNs	30wt.%	6.71	yes	yes
[154]	PVA	*h*-BN/MFC	30wt.%	4.61	yes	yes
[155]	PVA	*h*-BN	1.6 wt.%	0.26	yes	
[156]	PVA	*h*-BN	27 vol.%	8.44		yes
[157]	PVA	SiO_2@exfoliated *h*-BN	15.2wt.%	13.88	yes	yes
[158]	PVA	BNNT	5%	0.95	yes	
[159]	PVA+polyacrylamide	*h*-BN	20vol.%	0.47	yes	
[160]	PVA	*h*-BN	10%	0.7328	yes	
[161]	PVA	*h*-BN	90 wt.%	120	yes	yes
[162]	PVA	*h*-BN	0.8 wt.%	9.90	yes	yes
[163]	PVA	*h*-BN	15wt.%	9.77		yes
[164]	PVA	*h*-BN	30wt.%	4.3	yes	
[165]	PP	*h*-BN	25%	0.57	yes	
[166]	PP	*h*-BN	50wt.%	2.882		not sure
[167]	PP	*h*-BN	30%	0.47		
[168]	PP	*h*-BN	30%	0.512		
[169]	PP	*h*-BN	20vol.%	1.71		yes
[170]	PP	BN particles	9wt.%	0.46		
[171]	PP/PS blends	*h*-BN	14.5 wt.%	0.43	yes	
[172]	PP	*h*-BN	30wt.%	0.72	yes	
[173]	PP	*h*-BN	1%	0.398	yes	
[174]	PP	*h*-BNs	5 vol.%	0.41		
[175]	PP/PS	BN CF	14.5 wt.% BN and 18 wt.% CF	0.62		
[176]	PP	*h*-BN	20%	0.9395		
[177]	PP	*h*-BN	20%	0.68	yes	
[178]	PP	*h*-BN	25%	0.576	yes	
[179]	polyurethane (PU)	spherical *h*-BN@PMMA (SBp)	30wt.%	7.3		
[180]	PU	MoS_2/*h*-BN	0.5wt.%	0.92		yes
[181]	Thermoplastic polyurethane (TPU)	*h*-BN	10 wt.%	14.7		yes
[182]	TPU	BNNT	1.0 wt %	14.5		yes
[183]	PU	*h*-BN	40wt.%	10		not sure
[184]	TPU	*h*-BN	95wt.%	50.3		yes
[185]	TPU	*h*-BN	40wt.%	2.56		yes
[186]	TPU	BNNT	27.4wt.%	5.67	may be	yes
[187]	PU	*h*-BN	5wt.%	22		yes
[188]	PU	*h*-BN	30 wt.%	0.72	yes	
[189]	TPU	*h*-BNs	30 wt.%	5.15	yes	yes
[190]	PLA/TPU	*h*-BN	25%	0.69		
[191]	TPU	BN powders	50 wt.%	3.06		yes
[163]	TPU	*h*-BN	16wt.%	8.35		yes

[192]	Silicone rubber	*h*-BN	14wt.%	0.74		yes
[193]	Silicone rubber	*h*-BN nanoflake	40wt.%	0.901	yes	yes
[194]	Silicon rubber	GNP+BN	17.88vol.%	8.45		yes
[195]	Silicone rubber	BN particles	45 wt.%	0.745	yes	
[196]	Silicone	*h*-BN	12wt.%	0.36	yes	
[197]	Silicone rubber	BN	39vol.%	5.4		yes
[198]	Silicone thermal pad (STP)	*h*-BNs+Al_2O_3	10 wt.%	5.58		
[199]	Silicone rubber	boron nitride (BN) and aluminum nitride (AlN)	h-BN filler was 50 wt.%	0.554		
[200]	Silicone	BN	50 wt.%.	0.8837		
[201]	Silicone rubber	SiCw /*h*-BN	50 vol.%	1.28		not sure
[202]	Silicone rubber	*h*-BN	2%	0.24	yes	
[203]	Polyamide (PA)	*h*-BN	43.5vol.%	2		
[204]	Polyamide 6 (PA6)	*h*-BN	40vol.%	5.701		yes
[205]	PA 6	*h*-BN	30 vol.%	2.03		yes
[206]	PA 6	*h*-BN	40 vol.%	2.87		yes
[207]	PA 6	*h*-BN	40 wt.%	2.496		not sure
[208]	nylon 66	3D BN	15vol.%	0.85		
[209]	PA 12	BN	40 wt %	0.55		
[210]	PA6	BN@PDA@AgNPs	9.2 vol.%	0.673		
[211]	PA6	*h*-BN/graphene	1.6 wt.% *h*-BN/ 6.8 wt.% graphene	0.891		
[212]	PA	*h*-BN	30%	0.696	yes	yes
[213]	Nylon 12	BN	40%	0.55	yes	
[214]	PA 6	Modified *h*-BN	5%	0.498	yes	
[215]	poly (vinylidene fluoride) (PVDF)	*h*-BN	60wt.%	1.34		yes
[216]	PVDF	*h*-BN	30wt.%	7.29	yes	yes
[217]	PVDF	*h*-BN	60 wt.%	11.88		yes
[218]	PVDF	BN	30 wt.%	3.5		yes
[219]	PVDF	*h*-BN	33 wt %	16.3		yes
[220]	PVDF	*h*-BN/CNT	25 vol %	1.8	yes	
[221]	PVDF	*h*-BN	4 wt.%	4.69		yes
[222]	PVDF	*h*-BN	30wt.%	7.29	yes	yes
[223]	PVDF	*h*-BN	20wt.%	1.43	yes	yes
[224]	PVDF	Oriented boron nitride	10%	0.57		yes
[225]	PVDF	CNTs/BN	30 wt.%	0.73		
[226]	Cellulose nanofiber	*h*-BN	9.51vol.% /23.08wt.%	1.49		yes
[227]	Cellulose nanofiber	*h*-BN	1.0 wt.%	6.88	yes	yes
[228]	Cellulose nanofiber	BNNT	25wt.%	21.39		yes
[229]	Cellulose nanofiber	*h*-BN	25wt.%	22.67	yes	yes
[230]	Cellulose nanofiber	*h*-BNs	60wt.% EOH-*h*-BN	24.27	yes	yes
[231]	Cellulose nanofiber	*h*-BN	50 wt.%	24.66		yes
[232]	Cellulose nanofiber	*h*-BN	70wt.%	30.25	yes	yes
[233]	Cellulose nanofiber /na diamond	*h*-BN	44.3vol.%	6.17		yes
[234]	Nanofibrillated cellulose	BNNP	40wt.%	20.64	yes	yes

[235]	Nano fibrillated cellulose	*h*-BN	50 wt.%	65.7	可能	yes
[236]	Polymethyl methacrylate (PMMA)	VGs-*h*-BNs-VGs	29.3vol.%	4.03		yes
[237]	PMMA	*h*-BN powder	70 wt.%	3.73	yes	not sure
[238]	PMMA	*h*-BN	50 wt.%	7.3	yes	yes
[239]	PMMA	*h*-BN FOAM	0.07 vol.%	0.97		
[240]	PMMA	*h*-BN	14wt.%	1.21		not sure
[241]	PMMA	*h*-BN	10 wt.%	0.49		
[242]	PMMA	AgNP/*h*-BNs	35vol %	1.48	yes	not sure
[243]	PMMA	SiO2@BN	40 vol.%	5.583	yes	
[244]	Rubber	polyrhodanine@*h*-BNs (PR*h*-*h*-BNs)	27.5vol.%	1.5	yes	yes
[245]	Rubber	Graphite/BN	40wt.%	0.4		
[246]	Rubber	BN	40wt.%	1.37		
[247]	Rubber	*h*-BNs/polyrhodanine	77wt.%	45.7	yes	yes
[248]	Rubber	*h*-BN	40 vol.%,	1.110		not sure
[249]	Rubber	modification of boron nitride	30vol.%	0.39	yes	
[250]	Rubber	*h*-BN	40wt.%	1.8		yes
[251]	Rubber	boron nitride (BN)	18vol.%	1.179		yes
[252]	Rubber	boron nitride nanosheets (*h*-BNs)	10.5 vol.%	0.57		
[253]	Polyethylene glycol (PEG)	rGO/BN	18mg/ml	0.79		
[254]	PEG	*h*-BN	27 wt.%	2.77	yes	
[255]	PEG	boron nitride (BN)	30wt.%	3		
[256]	PEG	BN	30 wt %	3	yes	
[257]	PEG	Boron nitride	30wt.%	1.33		not sure
[258]	PEG	BN	30wt.%	2.15	yes	
[259]	Polyphenylene sulfide (PPS)	*h*-BN	40vol.%	4.15	yes	
[260]	PPS	carbon nanotube (CNT)@BN	40wt.%	2.39	yes	
[261]	PPS	*h*-BN	38wt.%	2.1		yes
[262]	PPS	*h*-BN	30vol.%	3.2		
[263]	PPS	*h*-BNs-MWCNT	50wt.%	6.3	yes	yes
[264]	PPS	surface-modified BN-Si	60 wt.%	3.09	yes	可能 yes
[265]	PPS	micrometer boron nitride/nanometer boron nitride	60 wt.%	2.638		not sure
[266]	Poly(arylene ether nitrile) (PEN)	*h*-BN/AgNPs	30wt.%	0.921	yes	
[267]	PEN	*h*-BN	16%	0.74	yes	
[268]	PEN	*h*-BN	30wt.%	0.662	yes	yes
[269]	PEN	*h*-BN	16wt.%	0.74	yes	not sure
[270]	PEN	*h*-BN	5%	0.47	yes	
[271]	PEN	BN	50 wt.%	1.63	yes	yes

[272]	Polystyrene (PS)/ CNT	*h*-BN	10vol.%	0.68		
[273]	PS	*h*-BN	50 wt.%	5.57		
[274]	PS	BN particles	33.3 wt %	0.94		
[275]	PS	*h*-BN	13.4 vol %	8	yes	yes
[276]	PS	*h*-BN	40wt.%	2.0	yes	yes
[277]	Aramid	*h*-BN	50%	0.6156	yes	
[278]	Aramid	*h*-BN@PDA	70wt.%	1.36	yes	not sure
[279]	Aramid	*h*-BN	70wt.%%	122.5		yes
[280]	Acrylonitrile Butadiene Styrene (ABS)	BN	35 wt.%	1.45		yes
[281]	ABS	boron nitride	20 wt.%	0.501	yes	
[282]	ABS	*h*-BN	20%	0.501	yes	
[283]	Polycarbonate (PC)	BN plates	18.5vol.%	3.09		yes
[284]	PC	cBN	20%	0.7341	yes	
[285]	PC	*h*-BN	20%	0.73413	yes	
[286]	Paraffin	*h*-BN	40wt.%	3.47		
[287]	Polylactide (PLA)	*h*-BN/LCP fiber	66.6vol.%	3.14		yes
[288]	PLA	*h*-BN/GNP	33.3 wt.%	2.77	yes	not sure
[289]	Polyamide-imide (PAI)	*h*-BN	9wt.%	7.69	yes	yes
[290]	PAI	*h*-BN	4%	1.17	yes	
[291]	Poly(3-hydroxybutyrate) (PHB)	*h*-BN	50wt.%	1.37		
[292]	PHB	*h*-BN/Al2O3	50wt.%	1.79		not sure
[293]	hybrid polymer	BN particles (3 μm)	20vol.%	0.58		
[294]	Hybrid polymer	*h*-BN	50vol.%	1.55	yes	
[295]	Perfluoroalkoxy	*h*-BN	30wt.%	4.65		yes
[296]	Poly(diallyl dimethyl ammonium chloride)	*h*-BN	90wt.%	212.8		yes
[297]	Poly(caprolactone)	*h*-BNs	20wt.%	1.96	yes	yes
[298]	Bismaleimide-triazine resin	*h*-BN	15wt.%%	0.63	yes	
[299]	Cyanate ester	BN-HBP	38wt.%	1.27	yes	
[300]	Slide-ring	BN particle	70wt.%	1.9	yes	
[301]	Polyether ether ketone	*h*-BN	5wt.%	0.363		
[302]	Polyphenylene ether resin	*h*-BN@SiO_2	48wt.%	1.08	yes	yes
[303]	Poly(fluorovinylidene-cohexafluoropropylene)	*h*-BN	1wt.%	0.91		
[304]	Benzoxazine	*h*-BN@PDA	20wt.%	0.71	yes	yes
[305]	Poly (2- ethylhexyl acrylate)	*h*-BN	30wt.%	4.2		yes
[306]	Polyvinylpyrrolidone nanofibers	BNNT	30wt.%	0.85	yes	yes
[307]	Ethylene-vinyl acetate copolymer	*h*-BN	50 wt.%	13.2	yes	yes
[308]	Epoxy polybutadien	*h*-BN	4 wt.%	0.187	yes	
[309]	Phosphorus-free bismaleimide resin	*h*-BN Skeleton	12.53 wt.%	1.53		
[310]	Polyphthalamide	*h*-BN	40wt.%	2.89	yes	yes
[311]	Poly(ε-caprolactone)/Poly(	*h*-BN	3 wt.%	0.8		

	lacticacid)					
[312]	Poly(ethylene oxide)	*h*-BN	71.4wt.%	3.4	yes	yes
[313]	Hybrid cyanate ester composites	BN	30wt.%	0.64		
[314]	Polybenzimidazole (OPBI)	*h*-BN	30 wt.%	8.58	yes	not sure
[315]	PAA	*h*-*h*-BN	50 wt %	3.5		not sure
[316]	Photopolymerizable	*h*-BN	35 wt.%	0.84	yes	
[317]	Phthalonitrile	na -BN	30 wt.%	4.69	yes	not sure
[318]	Polybutylene terephthalate	*h*-BN	80wt.%	15.1	yes	yes
[79]	polyurethane acrylate	BN–TiO_2	30vol.%	1.54	yes	
[319]	Styrene–ethylene–butylene–styrene	*h*-BN	95wt.%	45		yes
[320]	Bisphe l acyanate ester	*h*-BN	4%	0.63	yes	
[321]	poly(m-phenylene isophthalamide)	modified h-BN	30%	0.94<br>0.86	yes	
[322]	Polyphthalonitrile	functionalized *h*-BN	1:0.5	0.8	yes	
[323]	poly (2-ethylhexyl acrylate)	functionalized graphene (f-G) /BN	30wt.%	1.96	yes	yes
[324]	poly(m-phenyleneisophthal amide)	*h*-BN	12wt.%	8.06	yes	yes
[325]	n–octadecane (ODE) and stearic acid (SA) eutectics	*h*-BN	5wt.%	0.317		
[326]	Poly(hexahydrotriazine)	*h*-BN	55 vol %	14	yes	yes
[327]	Polyhydroxyalka ates	*h*-BN	2 wt.%	0.174		
[328]	Polybenzoxazine	micrometer boron nitride/nanometer boron nitride	25wt.%	0.9196		
[329]	Poly(styrene-acrylic acid)	*h*-BN	33.3%	0.877	yes	
[330]	Polycaprolactonegrafted polyrotaxane	*h*-BN	70wt.%	1.8	yes	not sure

4. References

[1] Permal A, Devarajan M, Hung HL, Zahner T, Lacey D, Ibrahim K. Thermal and mechanical properties of epoxy composite filled with binary particle system of polygonal aluminum oxide and boron nitride platelets. Journal of Materials Science 2016;51:7415-26.

[2] Wang ZD, Liu JY, Cheng YH, Chen SY, Yang MM, Huang JL, et al. Alignment of Boron Nitride Nanofibers in Epoxy Composite Films for Thermal Conductivity and Dielectric Breakdown Strength Improvement. Nanomaterials 2018;8.

[3] Abbasi S, Aravamudhan S, Ieee. Effect of Boron Nitride (hBN) Filler on Thermal Properties of Underfill Epoxy. Proceedings of the Sixteenth Intersociety Conference on Thermal and Thermomechanical Phenomena in Electronic Systems Itherm 20172017. p. 251-9.

[4] Li Z, Lira SIM, Zhang L, Exposito DF, Heeralal VB, Wang D-Y. Bio-inspired engineering of boron nitride with iron-derived nanocatalyst toward enhanced fire retardancy of epoxy resin. Polymer Degradation and Stability 2018;157:119-30.

[5] Liu X, Xiao M, Du B, Chen L, Zhang JW, Gong Z, et al. Thermal and Electrical Properties of Nanoparticle Oriented Epoxy/BN/SiC Composites for Superconducting Magnet. Ieee Transactions on Applied Superconductivity 2019;29.

[6] Yetgin H, Veziroglu S, Aktas OC, Yalcinkaya T. Enhancing thermal conductivity of epoxy with a binary filler system of h-BN platelets and Al2O3 nanoparticles. International Journal of Adhesion and Adhesives 2020;98.

[7] Lee J-H, Shin H, Rhee KY. Surface functionalization of boron nitride platelets via a catalytic oxidation/silanization process and thermomechanical properties of boron nitride epoxy composites. Composites Part B-Engineering 2019;157:276-82.

[8] Salehirad M, Nikje MMA, Ahmadian-Alam L. Synthesis and Characterization of Functionalized Fe3O4/Boron Nitride as Magnetically Alignable 2D-Nanofiller to Improve the Thermal Conductivity of Epoxy Nanocomposites. Industrial & Engineering Chemistry Research 2018;57:1803-14.

[9] 杜伯学, 杜强, 李进, 李昂, 傅明利, 肖微. 气体绝缘输电管道用环氧树脂/氮化硼高导热复合材料表面电荷动态特性. 高电压技术 2018;44:2646-53.

[10] Sangermano M, Razza N, Graham G, Barandiaran I, Kortaberria G. Electrically insulating polymeric nanocomposites with enhanced thermal conductivity by visible-light curing of epoxy-boron nitride nanotube formulations. Polymer

International 2017;66:1935-9.
[11] Agrawal R, Hanna J, Gunduz IE, Luhrs CC. Epoxy-PCM Composites with Nanocarbons or Multidimensional Boron Nitride as Heat Flow Enhancers. Molecules 2019;24.
[12] Weng L, Wang H, Zhang X, Liu L, Zhang H. Improved Thermal Conductivities of Epoxy Resins Containing Surface Functionalized BN Nanosheets. Nano 2018;13:1850133.
[13] Xu C, Qu T, Zhang X, Qu X, Wang N, Zhang Q, et al. Enhanced toughness and thermal conductivity for epoxy resin with a core-shell structured polyacrylic modifier and modified boron nitride. Rsc Advances 2019;9:8654-63.
[14] Yang H, Chen Q, Wang X, Chi M, Liu H, Ning X. Dielectric and Thermal Conductivity of Epoxy Resin Impregnated Nano-h-BN Modified Insulating Paper. Polymers 2019;11.
[15] Chen H, Zhao C, Xu S, Yang X, Iop. Preparation and Properties Study of Thermally Conductive Epoxy/Modified Boron Nitride/Graphene Nanosheets Composites. 1st International Conference on Frontiers of Materials Synthesis and Processing2017.
[16] 陈和祥, 赵春宝, 徐随春, 杨绪杰. 环氧树脂/氮化硼/石墨烯纳米片导热复合材料的制备与性能研究. 功能材料 2017;48:12064-8.
[17] 林美燕. 纳米氮化硼的制备及其在导热纸基覆铜板中的应用 [硕士]: 华南理工大学; 2019.
[18] 徐晨, 武向南, 张庆新, 瞿雄伟. 增韧导热环氧树脂/氮化硼复合材料的制备与表征. 化工进展 2018;37:4752-7.
[19] Zhang Y, Choi JR, Park S-J. Thermal conductivity and thermo-physical properties of nanodiamond-attached exfoliated hexagonal boron nitride/epoxy nanocomposites for microelectronics. Composites Part a-Applied Science and Manufacturing 2017;101:227-36.
[20] Lei Y, Han Z, Ren D, Pan H, Xu M, Liu X. Design of h-BN-Filled Cyanate/Epoxy Thermal Conductive Composite with Stable Dielectric Properties. Macromolecular Research 2018;26:602-8.
[21] 胡冰, 梁兵. 氮化硼纳米片的修饰及其在导热复合材料中的应用. 当代化工 2019;48:1909-13+25.
[22] Permal A, Devarajan M, Huong LH, Zahner T, Lacey D, Ibrahim K. Enhanced

Thermal and Mechanical Properties of Epoxy Composites Filled With Hybrid Filler System of Aluminium Nitride and Boron Nitride. Polymer Composites 2018;39:E1372-E80.
[23] Guo Y, He J, Wang H, Su Z, Qu Q, Tian R, et al. Boron Nitride-Graphene Sponge as Skeleton Filled With Epoxy Resin for Enhancing Thermal Conductivity and Electrical Insulation. Polymer Composites 2019;40:E1600-E11.
[24] Han Q, Zhang J, Wang XG. Enhanced through-thickness thermal conductivity of epoxy with cellulose-supported boron nitride nanosheets. Polymer 2019;179.
[25] 马万里，田付强，熊雯雯，姚江艺. 环氧树脂/氮化硼微纳米复合材料的导热与电气绝缘性能研究. 绝缘材料 2019;52:36-42.
[26] Weng L, Wang H, Zhang X, Liu L, Zhang H. Preparation and properties of boron nitride/epoxy composites with high thermal conductivity and electrical insulation. Journal of Materials Science-Materials in Electronics 2018;29:14267-76.
[27] 石倩. 氮化硼/环氧树脂导热复合材料的制备与性能研究 [硕士]: 贵州大学; 2019.
[28] 徐随春，赵春宝. 环氧树脂/改性氮化硼导热复合材料的制备与性能研究. 绝缘材料 2017;50:16-20.
[29] Mi Y, Gou JX, Liu LL, Ge X, Wan H, Liu Q. Enhanced Breakdown Strength and Thermal Conductivity of BN/EP Nanocomposites with Bipolar Nanosecond Pulse DBD Plasma Modified BNNSs. Nanomaterials 2019;9.
[30] Qu TG, Yang N, Hou J, Li GH, Yao YM, Zhang QX, et al. Flame retarding epoxy composites with poly(phosphazene-co-bisphenol A)-coated boron nitride to improve thermal conductivity and thermal stability. Rsc Advances 2017;7:6140-51.
[31] Ma JR, Luo N, Xie ZL, Chen F, Fu Q. Preparation of modified hexagonal boron nitride by ball-milling and enhanced thermal conductivity of epoxy resin. Materials Research Express 2019;6.
[32] Wang XW, Wu PY. Melamine foam-supported 3D interconnected boron nitride nanosheets network encapsulated in epoxy to achieve significant thermal conductivity enhancement at an ultralow filler loading. Chemical Engineering Journal 2018;348:723-31.
[33] Kim Y, Oh H, Kim J. Enhanced thermal conductivity of epoxy composites using boron nitride nanoplatelets prepared by Fe3O4 assisted liquid-phase exfoliation. Ceramics International 2019;45:24121-6.

[34] Zhang Y, Gao W, Li Y, Zhao D, Yin H. Hybrid fillers of hexagonal and cubic boron nitride in epoxy composites for thermal management applications. Rsc Advances 2019;9:7388-99.
[35] Gong Y, Zhou W, Kou Y, Xu L, Wu H, Zhao W. Heat conductive h-BN/CTPB/epoxy with enhanced dielectric properties for potential high-voltage applications. High Voltage 2017;2:172-8.
[36] Owais M, Zhao J, Imani A, Wang G, Zhang H, Zhang Z. Synergetic effect of hybrid fillers of boron nitride, graphene nanoplatelets, and short carbon fibers for enhanced thermal conductivity and electrical resistivity of epoxy nanocomposites. Composites Part a-Applied Science and Manufacturing 2019;117:11-22.
[37] Yang F, Sun X, Guo Q, Yao Z. Improvement of Thermal Conductivities for Epoxy Composites via Incorporating Poly(vinyl benzal)-Coated h-BN Fillers and Solvent-Assisted Dispersion. Industrial & Engineering Chemistry Research 2019;58:18635-43.
[38] 马振宁, 钟博, 王培侨, 王逊, 汪青杰. 氮化硼/环氧树脂绝缘导热材料的制备及性能表征. 材料导报 2016;30:65-9.
[39] Isarn I, Ramis X, Ferrando F, Serra A. Thermoconductive Thermosetting Composites Based on Boron Nitride Fillers and Thiol-Epoxy Matrices. Polymers 2018;10.
[40] Su Z, Wang H, He J, Guo YL, Qu QQ, Tian XY. Fabrication of Thermal Conductivity Enhanced Polymer Composites by Constructing an Oriented Three-Dimensional Staggered Interconnected Network of Boron Nitride Platelets and Carbon Nanotubes. Acs Applied Materials & Interfaces 2018;10:36342-51.
[41] Mi Y, Liu LL, Gui L, Ge X. Effect of frequency of microsecond pulsed electric field on orientation of boron nitride nanosheets and thermal conductivity of epoxy resin-based composites. Journal of Applied Physics 2019;126.
[42] Ribeiro H, Trigueiro JPC, Silva WM, Woellner CF, Owuor PS, Cristian Chipara A, et al. Hybrid MoS2/h-BN Nanofillers As Synergic Heat Dissipation and Reinforcement Additives in Epoxy Nanocomposites. ACS Appl Mater Interfaces 2019;11:24485-92.
[43] Yang XT, Zhu JH, Yang D, Zhang JL, Guo YQ, Zhong X, et al. High-efficiency improvement of thermal conductivities for epoxy composites from synthesized liquid crystal epoxy followed by doping BN fillers. Composites Part B-Engineering

2020;185.
[44] Sun JJ, Wang D, Yao YM, Zeng XL, Pan GR, Huang Y, et al. Boron nitride microsphere/epoxy composites with enhanced thermal conductivity. High Voltage 2017;2:147-53.
[45] Li X, Feng Y, Chen C, Ye Y, Zeng H, Qu H, et al. Highly thermally conductive flame retardant epoxy nanocomposites with multifunctional ionic liquid flame retardant-functionalized boron nitride nanosheets. Journal of Materials Chemistry A 2018;6:20500-12.
[46] Pawelski-Hoell C, Bhagwat S, Altstadt V. Thermal, fire, and mechanical properties of solvent-free processed BN/boehmite-filled prepregs. Polymer Engineering and Science 2019;59:1840-52.
[47] Yang X, Guo Y, Luo X, Zheng N, Ma T, Tan J, et al. Self-healing, recoverable epoxy elastomers and their composites with desirable thermal conductivities by incorporating BN fillers via in-situ polymerization. Composites Science and Technology 2018;164:59-64.
[48] Kim Y, Kim J. Fabrication of Fe3O4 coated boron nitride nanoplatelets by liquid-phase exfoliation for thermally enhanced epoxy composites via magnetic alignment. Composites Science and Technology 2020;188.
[49] Isarn I, Bonnaud L, Massagues L, Serra A, Ferrando F. Study of the synergistic effect of boron nitride and carbon nanotubes in the improvement of thermal conductivity of epoxy composites. Polymer International 2020;69:280-90.
[50] Chen C, Xue Y, Li Z, Wen Y, Li X, Wu F, et al. Construction of 3D boron nitride nanosheets/silver networks in epoxy-based composites with high thermal conductivity via in-situ sintering of silver nanoparticles. Chemical Engineering Journal 2019;369:1150-60.
[51] Bian W, Yao T, Chen M, Zhang C, Shao T, Yang Y. The synergistic effects of the micro-BN and nano-Al2O3 in micro-nano composites on enhancing the thermal conductivity for insulating epoxy resin. Composites Science and Technology 2018;168:420-8.
[52] Jiang Y, Shi X, Feng Y, Li S, Zhou X, Xie X. Enhanced thermal conductivity and ideal dielectric properties of epoxy composites containing polymer modified hexagonal boron nitride. Composites Part A: Applied Science and Manufacturing 2018;107:657-64.
[53] Minh Canh V, Tuan Sang T, Bae YH, Yu MJ, Vu Chi D, Lee JH, et al.

Self-Assembly of Carbon Nanotubes and Boron Nitride via Electrostatic Interaction for Epoxy Composites of High Thermal Conductivity and Electrical Resistivity. Macromolecular Research 2018;26:521-8.

[54] Su Z, Wang H, Ye X, Tian K, Huang W, He J, et al. Anisotropic thermally conductive flexible polymer composites filled with hexagonal born nitride (h-BN) platelets and ammine carbon nanotubes (CNT-NH2): Effects of the filler distribution and orientation. Composites Part a-Applied Science and Manufacturing 2018;109:402-12.

[55] Su Z, Wang H, Ye X, Tian K, Huang W, He J, et al. Synergistic enhancement of anisotropic thermal transport flexible polymer composites filled with multi-layer graphene (mG) and mussel-inspiring modified hexagonal boron nitride (h-BN). Composites Part a-Applied Science and Manufacturing 2018;111:12-22.

[56] 陈赟, 杨威, 张翀, 张卓, 陈新, 边凯, et al. 高导热氧化铝/氮化硼环氧复合绝缘材料性能研究. 化学与黏合 2018;40:243-6.

[57] Agrawal A, Chandrakar S. Influence of particulate surface treatment on physical, mechanical, thermal, and dielectric behavior of epoxy/hexagonal boron nitride composites. Polymer Composites.

[58] Kim K, Ju H, Kim J. Surface modification of BN/Fe3O4 hybrid particle to enhance interfacial affinity for high thermal conductive material. Polymer 2016;91:74-80.

[59] Zhang C, Huang R, Wang Y, Wu Z, Guo S, Zhang H, et al. Aminopropyltrimethoxysilane-functionalized boron nitride nanotube based epoxy nanocomposites with simultaneous high thermal conductivity and excellent electrical insulation. Journal of Materials Chemistry A 2018;6:20663-8.

[60] Zhang H, Huang R, Li Y, Li H, Wu Z, Huang J, et al. Optimization of boron nitride sphere loading in epoxy: enhanced thermal conductivity and excellent electrical insulation. Polymers 2019;11:1335.

[61] Lee D, Lee S, Byun S, Paik K-W, Song SH. Novel dielectric BN/epoxy nanocomposites with enhanced heat dissipation performance for electronic packaging. Composites Part a-Applied Science and Manufacturing 2018;107:217-23.

[62] Li Y, Zhang H, Yang X, He G, Yang Z, Li J. The combustion synthesis of highly crystalline boron nitride nanosheets and their application in thermoconductive polymeric composites. Crystengcomm 2019;21:5461-9.

[63] Leng X, Xiao C, Chen L, Su Z, Zheng K, Zhang X, et al. An efficient approach for constructing 3-D boron nitride networks with epoxy composites to form materials with enhanced thermal, dielectric, and mechanical properties. High Performance Polymers 2019;31:350-8.
[64] 冷鑫钰, 肖超, 陈璐, 郑康, 张献, 田兴友. 环氧树脂中 3D 氮化硼复合导热网络的构筑及性能. 高分子材料科学与工程 2019;35:102-7.
[65] Kumar R, Nayak SK, Sahoo S, Panda BP, Mohanty S, Nayak SK. Study on thermal conductive epoxy adhesive based on adopting hexagonal boron nitride/graphite hybrids. Journal of Materials Science-Materials in Electronics 2018;29:16932-8.
[66] 王志晓. 环氧树脂/氮化硼高导热复合材料的制备与性能研究 [硕士]: 北京交通大学; 2019.
[67] Jung DW, Kim JM, Yoon HW, Nam KM, Kwon YE, Jeong S, et al. Solution-processable thermally conductive polymer composite adhesives of benzyl-alcohol-modified boron nitride two-dimensional nanoplates. Chemical Engineering Journal 2019;361:783-91.
[68] Wu YJ, Zhang XX, Negi A, He JX, Hu GX, Tian SS, et al. Synergistic Effects of Boron Nitride (BN) Nanosheets and Silver (Ag) Nanoparticles on Thermal Conductivity and Electrical Properties of Epoxy Nanocomposites. Polymers 2020;12.
[69] Isarn I, Bonnaud L, Massagues L, Serra A, Ferrando F. Enhancement of thermal conductivity in epoxy coatings through the combined addition of expanded graphite and boron nitride fillers. Progress in Organic Coatings 2019;133:299-308.
[70] Qin TF, Wang H, He J, Wang R, Qu QQ, Tian XY. Preparation and application of high thermal conductivity TMBPDGE-DDM@h-BN composites. Materials Research Express 2019;6.
[71] Huang T, Zeng XL, Yao YM, Sun R, Meng FL, Xu JB, et al. Boron nitride@graphene oxide hybrids for epoxy composites with enhanced thermal conductivity. Rsc Advances 2016;6:35847-54.
[72] Zhang RH, Shi XT, Tang L, Liu Z, Zhang JL, Guo YQ, et al. Thermally Conductive and Insulating Epoxy Composites by Synchronously Incorporating Si-sol Functionalized Glass Fibers and Boron Nitride Fillers. Chinese Journal of Polymer Science.
[73] Zou D, Huang X, Zhu Y, Chen J, Jiang P. Boron nitride nanosheets endow the

traditional dielectric polymer composites with advanced thermal management capability. Composites Science and Technology 2019;177:88-95.
[74] Chung SL, Lin JS. Thermal Conductivity of Epoxy Resin Composites Filled with Combustion Synthesized h-BN Particles. Molecules 2016;21.
[75] Hutchinson JM, Roman F, Cortes P, Calventus Y. Epoxy composites filled with boron nitride and aluminum nitride for improved thermal conductivity. Polimery 2017;62:560-6.
[76] Jakubinek MB, Niven JF, Johnson MB, Ashrafi B, Kim KS, Simard B, et al. Thermal conductivity of bulk boron nitride nanotube sheets and their epoxy-impregnated composites. Physica Status Solidi a-Applications and Materials Science 2016;213:2237-42.
[77] Wang FF, Zeng XL, Yao YM, Sun R, Xu JB, Wong CP. Silver Nanoparticle-Deposited Boron Nitride Nanosheets as Fillers for Polymeric Composites with High Thermal Conductivity. Scientific Reports 2016;6.
[78] Chen J, Huang X, Zhu Y, Jiang P. Cellulose nanofiber supported 3d interconnected bn nanosheets for epoxy nanocomposites with ultrahigh thermal management capability. Advanced Functional Materials 2017;27:1604754.
[79] Kim K, Kim J. Vertical filler alignment of boron nitride/epoxy composite for thermal conductivity enhancement via external magnetic field. International Journal of Thermal Sciences 2016;100:29-36.
[80] Huang T, Yao YM, Meng FL. GRAPHENE ENCAPSULATING BORON NITRIDE ELECTROSTATIC ASSEMBLIES FOR FABRICATION OF POLYMER COMPOSITES WITH HIGH THERMAL CONDUCTIVITY. Advanced Composites Letters 2016;25:147-50.
[81] Huang T, Zeng X, Yao Y, Sun R, Meng F, Xu J, et al. A novel h-BN-RGO hybrids for epoxy resin composites achieving enhanced high thermal conductivity and energy density. Rsc Advances 2017;7:23355-62.
[82] Jang I, Shin KH, Yang I, Kim H, Kim J, Kim WH, et al. Enhancement of thermal conductivity of BN/epoxy composite through surface modification with silane coupling agents. Colloids and Surfaces a-Physicochemical and Engineering Aspects 2017;518:64-72.
[83] Kim K, Ju H, Kim J. Pyrolysis behavior of polysilazane and polysilazane-coated-boron nitride for high thermal conductive composite. Composites Science and Technology 2017;141:1-7.

[84] Tang L, He MK, Na XY, Guan XF, Zhang RH, Zhang JL, et al. Functionalized glass fibers cloth/spherical BN fillers/epoxy laminated composites with excellent thermal conductivities and electrical insulation properties. Composites Communications 2019;16:5-10.

[85] Hutchinson JM, Roman F, Folch A. Epoxy-Thiol Systems Filled with Boron Nitride for High Thermal Conductivity Applications. Polymers 2018;10.

[86] Hu J, Zeng X, Sun R, Xu J-b, Wong C-p, Ieee. Hierarchically Interconnected Epoxy/BN-SCMC Polymer Composites with Enhanced Thermal Conductivity2017.

[87] Moradi S, Calventus Y, Roman F, Hutchinson JM. Achieving High Thermal Conductivity in Epoxy Composites: Effect of Boron Nitride Particle Size and Matrix-Filler Interface. Polymers 2019;11.

[88] Van-Dung M, Lee D-I, Park J-H, Lee D-S. Rheological Properties and Thermal Conductivity of Epoxy Resins Filled with a Mixture of Alumina and Boron Nitride. Polymers 2019;11.

[89] Hu JT, Huang Y, Yao YM, Pan GR, Sun JJ, Zeng XL, et al. Polymer Composite with Improved Thermal Conductivity by Constructing a Hierarchically Ordered Three-Dimensional Interconnected Network of BN. Acs Applied Materials & Interfaces 2017;9:13544-53.

[90] Zheng XR, Park CW. Thermal and mechanical properties of carbon fiber-reinforced resin composites with copper/boron nitride coating. Composite Structures 2019;220:494-501.

[91] Tian ZL, Sun JJ, Wang SG, Zeng XL, Zhou S, Bai SL, et al. A thermal interface material based on foam-templated three-dimensional hierarchical porous boron nitride. Journal of Materials Chemistry A 2018;6:17540-7.

[92] Kargar F, Barani Z, Salgado R, Debnath B, Lewis JS, Aytan E, et al. Thermal Percolation Threshold and Thermal Properties of Composites with High Loading of Graphene and Boron Nitride Fillers. Acs Applied Materials & Interfaces 2018;10:37555-65.

[93] Liu Z, Li JH, Liu XH. Novel Functionalized BN Nanosheets/Epoxy Composites with Advanced Thermal Conductivity and Mechanical Properties. Acs Applied Materials & Interfaces 2020;12:6503-15.

[94] Hu J, Huang Y, Zeng X, Li Q, Ren L, Sun R, et al. Polymer composite with enhanced thermal conductivity and mechanical strength through orientation manipulating of BN. Composites Science and Technology 2018;160:127-37.

[95] Xia CL, Garcia AC, Shi SQ, Qiu Y, Warner N, Wu YJ, et al. Hybrid boron nitride-natural fiber composites for enhanced thermal conductivity. Scientific Reports 2016;6.
[96] Lewis JS, Barani Z, Magana AS, Kargar F, Balandin AA. Thermal and electrical conductivity control in hybrid composites with graphene and boron nitride fillers. Materials Research Express 2019;6.
[97] Han JK, Du GL, Gao WW, Bai H. An Anisotropically High Thermal Conductive Boron Nitride/Epoxy Composite Based on Nacre-Mimetic 3D Network. Advanced Functional Materials 2019;29.
[98] Kim K, Ryu S, Kim J. Melt-processable aggregated boron nitride particle via polysilazane coating for thermal conductive composite. Ceramics International 2017;43:2441-7.
[99] Fan B, Liu Y, He D, Bai J. Enhanced thermal conductivity for mesophase pitch-based carbon fiber/modified boron nitride/epoxy composites. Polymer 2017;122:71-6.
[100] Yu CP, Zhang J, Li Z, Tian W, Wang LJ, Luo J, et al. Enhanced through-plane thermal conductivity of boron nitride/epoxy composites. Composites Part a-Applied Science and Manufacturing 2017;98:25-31.
[101] Wang FF, Yao YM, Zeng XL, Huang T, Sun R, Xu JB, et al. Highly thermally conductive polymer nanocomposites based on boron nitride nanosheets decorated with silver nanoparticles. Rsc Advances 2016;6:41630-6.
[102] Xiao C, Tang Y, Chen L, Zhang X, Zheng K, Tian X. Preparation of highly thermally conductive epoxy resin composites via hollow boron nitride microbeads with segregated structure. Composites Part a-Applied Science and Manufacturing 2019;121:330-40.
[103] Zhu Z, Wang P, Lv P, Xu T, Zheng J, Ma C, et al. Densely packed polymer/boron nitride composite for superior anisotropic thermal conductivity. Polymer Composites 2018;39:E1653-E8.
[104] Nagaoka S, Jodai T, Kameyama Y, Horikawa M, Shirosaki T, Ryu N, et al. Cellulose/boron nitride core-shell microbeads providing high thermal conductivity for thermally conductive composite sheets. Rsc Advances 2016;6:33036-42.
[105] 朱志佳. 高性能氮化硼/树脂导热复合材料的制备及其热管理应用的研究[硕士]: 南京邮电大学; 2017.

[106] Shi A, Li Y, Liu W, Lei J, Xu L, Li Z-M. Enhanced thermal conductivity of multilayered sheets of polyethylene and boron nitride via promoting molecular diffusion between layers. Journal of Applied Physics 2019;125.
[107] Zhao TB, Zhang XL. Enhanced thermal conductivity of PE/BN composites through controlling crystallization behavior of PE matrix. Polymer Composites 2017;38:2806-13.
[108] Yang SY, Huang YF, Lei J, Zhu L, Li ZM. Enhanced thermal conductivity of polyethylene/boron nitride multilayer sheets through annealing. Composites Part a-Applied Science and Manufacturing 2018;107:135-43.
[109] Xiong SW, Zhang P, Xia Y, Fu PG, Gai JG. Antimicrobial hexagonal boron nitride nanoplatelet composites for the thermal management of medical electronic devices. Materials Chemistry Frontiers 2019;3:2455-62.
[110] Ding H, Guo YT, Leung SN. Development of thermally conductive polymer matrix composites by foaming-assisted networking of micron- and submicron-scale hexagonal boron nitride. Journal of Applied Polymer Science 2016;133.
[111] Wang Z, Li Q, Chen Z, Li H, Zheng S, Tang X. Functionalization of boron nitride nanosheets by diazonium salt for preparation of nanocomposites with high-density polyethylene. Polymer Composites 2019;40:2346-56.
[112] Borjas-Ramos JJ, Ramos-de-Valle LF, Neira-Velazquez MG, Hernandez-Hernandez E, Saucedo-Salazar EM, Soria-Arguello G. Thermal Conductivity of Nanocomposites Based in High Density Polyethylene and Surface Modified Hexagonal Boron Nitride via Cold Ethylene Plasma. Plasma Chemistry and Plasma Processing 2018;38:429-41.
[113] Muratov DS, Stepashkin AA, Anshin SM, Kuznetsov DV. Controlling thermal conductivity of high density polyethylene filled with modified hexagonal boron nitride (hBN). Journal of Alloys and Compounds 2018;735:1200-5.
[114] Shi A, Li Y, Liu W, Xu J-Z, Yan D-X, Lei J, et al. Highly thermally conductive and mechanically robust composite of linear ultrahigh molecular weight polyethylene and boron nitride via constructing nacre-like structure. Composites Science and Technology 2019;184:107858.
[115] Wang X, Lu H, Feng CP, Ni HY, Chen J. Facile method to fabricate highly thermally conductive UHMWPE/BN composites with the segregated structure for thermal management. Plastics Rubber and Composites.
[116] Zhou T, Smith MK, Berenguer JP, Quill TJ, Cola BA, Kalaitzidou K, et al. The

impact of polymer matrix blends on thermal and mechanical properties of boron nitride composites. Journal of Applied Polymer Science 2019;137:48661.
[117] Hamidinejad M, Zandieh A, Lee JH, Papillon J, Zhao B, Moghimian N, et al. Insight into the Directional Thermal Transport of Hexagonal Boron Nitride Composites. Acs Applied Materials & Interfaces 2019;11:41726-35.
[118] Guo YY, Cao CL, Fb L, Huang BQ, Xiao LR, Qian QR, et al. Largely enhanced thermal conductivity and thermal stability of ultra high molecular weight polyethylene composites via BN/CNT synergy. Rsc Advances 2019;9:40800-9.
[119] Zhang XM, Zhang JJ, Xia LC, Li CH, Wang JF, Xu F, et al. Simple and Consecutive Melt Extrusion Method to Fabricate Thermally Conductive Composites with Highly Oriented Boron Nitrides. Acs Applied Materials & Interfaces 2017;9:22977-84.
[120] Gao CW, Lu H, Ni HY, Chen J. Structure, thermal conductive, dielectric and electrical insulating properties of UHMWPE/BN composites with a segregated structure. Journal of Polymer Research 2017;25.
[121] Ren PG, Si XH, Sun ZF, Ren F, Pei L, Hou SY. Synergistic effect of BN and MWCNT hybrid fillers on thermal conductivity and thermal stability of ultra-high-molecular-weight polyethylene composites with a segregated structure. Journal of Polymer Research 2016;23.
[122] Nie SQ, Zhang XL, Luo J, Liu Y, Yan W. Synergistic effect of boron nitride and tetrapod-shaped zinc oxide whisker hybrid fillers on filler networks in thermal conductive HDPE composites. Polymer Composites 2017;38:1902-9.
[123] 杜伯学, 崔彬, 徐航, 李进, 傅明利, 侯帅. 聚乙烯/氮化硼高导热复合材料的耐电弧性和介电性能. 高电压技术 2018;44:1412-20.
[124] Zimmermann-Ptacek J, Muggli M, Wildhack S, Hintzer K, Gerdes T, Willert-Porada M, et al. Thermal, dielectric, and mechanical properties of h-BN-filled PTFE composites. Journal of Applied Polymer Science 2018;135.
[125] Pan C, Kou KC, Zhang Y, Li ZY, Wu GL. Enhanced through-plane thermal conductivity of PTFE composites with hybrid fillers of hexagonal boron nitride platelets and aluminum nitride particles. Composites Part B-Engineering 2018;153:1-8.
[126] Pan C, Zhang JQ, Kou KC, Zhang Y, Wu GL. Investigation of the through-plane thermal conductivity of polymer composites with in-plane oriented hexagonal boron

nitride. International Journal of Heat and Mass Transfer 2018;120:1-8.
[127] Cai XZ, Dong XZ, Lv WX, Ji CZ, Jiang ZY, Zhang XR, et al. Synergistic enhancement of thermal conductivity for low dielectric constant boron nitride-polytetrafluoroethylene composites by adding small content of graphene nanosheets. Composites Communications 2020;17:163-9.
[128] Gu J, Lv Z, Wu Y, Guo Y, Tian L, Qiu H, et al. Dielectric thermally conductive boron nitride/polyimide composites with outstanding thermal stabilities via in -situ polymerization-electrospinning-hot press method. Composites Part A: Applied Science and Manufacturing 2017;94:209-16.
[129] Haruki M, Tanaka K, Tada J, Onishi H, Tada Y. Effective thermal conductivity for nanocarbon/polyimide and carbon nanofiber/hexagonal boron nitride/polyimide composites. Polymer Composites 2018;40:3032-9.
[130] Guo Y, Lyu Z, Yang X, Lu Y, Ruan K, Wu Y, et al. Enhanced thermal conductivities and decreased thermal resistances of functionalized boron nitride/polyimide composites. Composites Part B: Engineering 2019;164:732-9.
[131] Liu L, Shen S, Wang Y. Enhanced thermal conductivity of flexible h-BN/polyimide composites films with ethyl cellulose. E-Polymers 2019;19:305-12.
[132] Park OK, Owuor PS, Jaques YM, Galvao DS, Kim NH, Lee JH, et al. Hexagonal boron nitride-carbon nanotube hybrid network structure for enhanced thermal, mechanical and electrical properties of polyimide nanocomposites. Composites Science and Technology 2020;188.
[133] Wang HT, Ding DL, Liu Q, Chen YH, Zhang QY. Highly anisotropic thermally conductive polyimide composites via the alignment of boron nitride platelets. Composites Part B-Engineering 2019;158:311-8.
[134] Wang T, Wang M, Fu L, Duan Z, Chen Y, Hou X, et al. Enhanced thermal conductivity of polyimide composites with boron nitride nanosheets. Scientific Reports 2018;8:1557.
[135] Zhou YC, Liu F. High-performance polyimide nanocomposites with core-shell AgNWs@BN for electronic packagings. Applied Physics Letters 2016;109.
[136] Yang LQ, Feng Y, Li ST, Xu CM, Chen G. Fabrication of h-BN nano-sheet composite and evaluation of microcosmic physical interfaces effect on thermal diffusion. Rsc Advances 2016;6:97969-75.
[137] Zhang G-D, Fan L, Bai L, He M-H, Zhai L, Mo S. Mesoscopic Simulation Assistant Design of Immiscible Polyimide/BN Blend Films with Enhanced Thermal

Conductivity. Chinese Journal of Polymer Science 2018;36:1394-402.
[138] Isarn I, Massagues L, Ramis X, Serra A, Ferrando F. New BN-epoxy composites obtained by thermal latent cationic curing with enhanced thermal conductivity. Composites Part a-Applied Science and Manufacturing 2017;103:35-47.
[139] Yang N, Xu C, Hou J, Yao YM, Zhang QX, Grami ME, et al. Preparation and properties of thermally conductive polyimide/boron nitride composites. Rsc Advances 2016;6:18279-87.
[140] Liu X, Ji T, Li N, Liu Y, Yin J, Su B, et al. Preparation of polyimide composites reinforced with oxygen doped boron nitride nano-sheet as multifunctional materials. Materials & Design 2019;180.
[141] Zhou Y, Yu S, Niu H, Liu F. Synergistic Improvement in Thermal Conductivity of Polyimide Nanocomposite Films Using Boron Nitride Coated Copper Nanoparticles and Nanowires. Polymers 2018;10.
[142] Zhang S, Li X, Guan X, Shi Y, Wu K, Liang L, et al. Synthesis of pyridine-containing diamine and properties of its polyimides and polyimide/hexagonal boron nitride composite films. Composites Science and Technology 2017;152:165-72.
[143] 杨曦, 姚亚超, 张伯涵, 苏创, 于晓燕. 高导热聚酰亚胺/氮化硼复合材料的制备与表征. 胶体与聚合物 2016;34:19-21.
[144] 杨娜, 王农跃, 姚艳梅, 潘滋涵, 瞿雄伟. 氮化硼和纳米金刚石混杂填充聚酰亚胺导热复合材料的制备与表征. 高分子材料科学与工程 2017;33:153-7.
[145] Liu LZ, Cao CH, Ma XY, Zhang XR, Lv T. Thermal conductivity of polyimide/AlN and polyimide/(AlN plus BN) composite films prepared by in-situ polymerization. Journal of Macromolecular Science Part a-Pure and Applied Chemistry 2020;57:398-407.
[146] Ma X, Liu L, Zhang X, Lv T. Thermal conductivity enhancement of polyimide films filled with BN and AlN fillers. High Performance Polymers 2019;31:959-68.
[147] Haruki M, Tada J, Funaki R, Onishi H, Tada Y. Enhancing thermal conductivities of hexagonal boron nitride/fluorinated polyimide composite materials using direct current electrical fields. Thermochimica Acta 2020;684.
[148] Kwon OH, Ha T, Kim DG, Kim BG, Kim YS, Shin TJ, et al. Anisotropy-Driven High Thermal Conductivity in Stretchable Poly(vinyl alcohol)/Hexagonal Boron Nitride Nanohybrid Films. Acs Applied Materials & Interfaces 2018;10:34625-33.

[149] Jing L, Li H, Tay RY, Sun B, Tsang SH, Cometto O, et al. Biocompatible Hydroxylated Boron Nitride Nanosheets/Poly(vinyl alcohol) Interpenetrating Hydrogels with Enhanced Mechanical and Thermal Responses. ACS nano 2017;11:3742-51.
[150] Li H, Gao Y, Zhu P, Du X, Yu X, Ma L, et al. Cationic Polyelectrolyte Bridged Boron Nitride Microplatelet Based Poly(vinyl alcohol) Composite: A Novel Method toward High Thermal Conductivity. Advanced Materials Interfaces 2019;6.
[151] Kim D, Ha S, Choi HK, Yu J, Kim YA. Chemical assembling of amine functionalized boron nitride nanotubes onto polymeric nanofiber film for improving their thermal conductivity. Rsc Advances 2018;8:4426-33.
[152] Prusty K, Swain SK. h-BN huddled starch reinforced polyethylhexylacrylate\polyvinyl alcohol thin films for packaging applications. Polymer Composites 2019;40:1810-8.
[153] Geng R-J, Song-Feng E, Li C-W, Li T-T, Wu J, Yao Y-G. High Crystallinity Boron Nitride Nanosheets: Preparation and the Property of BNNSs/Polyvinyl Alcohol Composite Film. Journal of Inorganic Materials 2019;34:401-6.
[154] Ge X, Liang W-J, Ge J-F, Chen X-J, Ji J-Y, Pang X-Y, et al. Hexagonal Boron Nitride/Microfibril Cellulose/Poly(vinyl alcohol) Ternary Composite Film with Thermal Conductivity and Flexibility. Materials 2019;12.
[155] Zhang J, Lei W, Chen J, Liu D, Tang B, Li J, et al. Enhancing the thermal and mechanical properties of polyvinyl alcohol (PVA) with boron nitride nanosheets and cellulose nanocrystals. Polymer 2018;148:101-8.
[156] Zhang J, Wang X, Yu C, Li Q, Li Z, Li C, et al. A facile method to prepare flexible boron nitride/poly(vinyl alcohol) composites with enhanced thermal conductivity. Composites Science and Technology 2017;149:41-7.
[157] Zhang J, Li C, Yu C, Wang X, Li Q, Lu H, et al. Large improvement of thermal transport and mechanical performance of polyvinyl alcohol composites based on interface enhanced by SiO2 nanoparticle-modified-hexagonal boron nitride. Composites Science and Technology 2019;169:167-75.
[158] Li CW, Long XY, E SF, Zhang QC, Li TT, Wu J, et al. Magnesium-induced preparation of boron nitride nanotubes and their application in thermal interface materials. Nanoscale 2019;11:11457-63.
[159] Xing L, Hu CX, Zhang YL, Wang XD, Shi LY, Ran R. A mechanically robust double-network hydrogel with high thermal responses via doping hydroxylated boron

nitride nanosheets. Journal of Materials Science 2019;54:3368-82.
[160] Yin C-G, Ma Y, Liu Z-J, Fan J-C, Shi P-H, Xu Q-J, et al. Multifunctional boron nitride nanosheet/polymer composite nanofiber membranes. Polymer 2019;162:100-7.
[161] Wang JM, Wu YP, Xue Y, Liu D, Wang XB, Hu X, et al. Super-compatible functional boron nitride nanosheets/polymer films with excellent mechanical properties and ultra-high thermal conductivity for thermal management. Journal of Materials Chemistry C 2018;6:1363-9.
[162] Zhang J, Lei WW, Liu D, Wang XG. Synergistic influence from the hybridization of boron nitride and graphene oxide nanosheets on the thermal conductivity and mechanical properties of polymer nanocomposites. Composites Science and Technology 2017;151:252-7.
[163] 耿仁杰. 高导热氮化硼纳米片的模板法制备及应用研究 [硕士]: 武汉科技大学; 2018.
[164] 刘群. 氮化硼修饰、导热复合材料和碳纤维氮化硼涂层的制备及性能研究 [硕士]: 山东大学; 2018.
[165] Chen L, Xu HF, He SJ, Du YH, Yu NJ, Du XZ, et al. Thermal Conductivity Performance of Polypropylene Composites Filled with Polydopamine-Functionalized Hexagonal Boron Nitride. Plos One 2017;12.
[166] Cheewawuttipong W, Tanoue S, Uematsu H, Iemoto Y. Thermal conductivity of polypropylene composites with hybrid fillers of boron nitride and vapor-grown carbon fiber. Polymer Composites 2016;37:936-42.
[167] 钟世龙. 氮化硼/碳纳米管及氮化硼/石墨烯的三维“桥联”导热网络在聚丙烯基体中的构筑 [硕士]: 西南大学; 2018.
[168] 徐鸿飞, 陈林, 俞南杰, 杜亦航, 林俊, 杜小泽. 氮化硼导热复合材料的制备与性能. 功能材料 2016;47:148-52.
[169] Zha XJ, Yang J, Pu JH, Feng CP, Bai L, Bao RY, et al. Enhanced Thermal Conductivity and Balanced Mechanical Performance of PP/BN Composites with 1 vol% Finely Dispersed MWCNTs Assisted by OBC. Advanced Materials Interfaces 2019;6.
[170] Du BX, Cui B. Effects of Thermal Conductivity on Dielectric Breakdown of Micro, Nano Sized BN Filled Polypropylene Composites. Ieee Transactions on Dielectrics and Electrical Insulation 2016;23:2116-25.
[171] Jiang X, Ma P, You F, Yao C, Yao J, Liu F. A facile strategy for modifying boron nitride and enhancing its effect on the thermal conductivity of

polypropylene/polystyrene blends. Rsc Advances 2018;8:32132-7.
[172] Zhong SL, Zhou ZY, Zhang K, Shi YD, Chen YF, Chen XD, et al. Formation of thermally conductive networks in isotactic polypropylene/hexagonal boron nitride composites via "Bridge Effect" of multi-wall carbon nanotubes and graphene nanoplatelets. Rsc Advances 2016;6:98571-80.
[173] Kong S, Seo H, Shin H, Baik J-H, Oh J, Kim Y-O, et al. Improvement in mechanical and thermal properties of polypropylene nanocomposites using an extremely small amount of alkyl chain-grafted hexagonal boron nitride nanosheets. Polymer 2019;180:121714.
[174] Seyhan AT, Goncu Y, Durukan O, Akay A, Ay N. Silanization of boron nitride nanosheets (BNNSs) through microfluidization and their use for producing thermally conductive and electrically insulating polymer nanocomposites. Journal of Solid State Chemistry 2017;249:98-107.
[175] Jiang XL, Ma PF, Zhou C, Zhu WW, You F, Yao C, et al. Simultaneously enhancing the thermal conductivity and dielectric constant of BN/CF hybrid filled polypropylene/polystyrene composites via in situ reactive processing. Polymer Composites.
[176] 杨帅, 敖玉辉, 肖凌寒. 聚丙烯/碳纤维/氮化硼导热绝缘复合材料的制备. 塑料工业 2017;45:129-32.
[177] 刘路. 聚丙烯/氮化硼导热绝缘复合材料的制备与性能 [硕士]: 武汉理工大学; 2016.
[178] 徐鸿飞. 聚丙烯/氮化硼导热复合材料的制备、性能及热导率模型研究 [硕士]: 华北电力大学(北京); 2017.
[179] Su K-H, Su C-Y, Cho C-T, Lin C-H, Jhou G-F, Chang C-C. Development of thermally conductive polyurethane composite by low filler loading of spherical BN/PMMA composite powder. Scientific Reports 2019;9.
[180] Ribeiro H, Trigueiro JPC, Lopes MC, Pedrotti JJ, Woellner CF, Silva WM, et al. Enhanced thermal conductivity and mechanical properties of hybrid MoS2/h-BN polyurethane nanocomposites. Journal of Applied Polymer Science 2018;135.
[181] Zhu Z, Li C, Songfeng E, Xie L, Geng R, Lin C-T, et al. Enhanced thermal conductivity of polyurethane composites via engineering small/large sizes interconnected boron nitride nanosheets. Composites Science and Technology 2019;170:93-100.

[182] Wang LJ, Han DB, Luo J, Li TT, Lin ZY, Yao YG. Highly Efficient Growth of Boron Nitride Nanotubes and the Thermal Conductivity of Their Polymer Composites. Journal of Physical Chemistry C 2018;122:1867-73.
[183] Hong H-J, Kwan SM, Lee DS, Kim SM, Kim YH, Lim JS, et al. Highly flexible and stretchable thermally conductive composite film by polyurethane supported 3D networks of boron nitride. Composites Science and Technology 2017;152:94-100.
[184] Yu C, Gong W, Tian W, Zhang Q, Xu Y, Lin Z, et al. Hot-pressing induced alignment of boron nitride in polyurethane for composite films with thermal conductivity over 50 Wm−1 K−1. Composites Science and Technology 2018;160:199-207.
[185] Liu J, Li W, Guo Y, Zhang H, Zhang Z. Improved thermal conductivity of thermoplastic polyurethane via aligned boron nitride platelets assisted by 3D printing. Composites Part A: Applied Science and Manufacturing 2019;120:140-6.
[186] Songfeng E, Geng R, Zhu Z, Xie L, Lu W, Li C, et al. Large-scale fabrication of boron nitride nanotubes and their application in thermoplastic polyurethane based composite for improved thermal conductivity. Ceramics International 2018;44:22794-9.
[187] Sadej M, Gierz L, Naumowicz M. Polyurethane composites with enhanced thermal conductivity containing boron nitrides. Polimery 2019;64:592-5.
[188] Ryu S, Oh H, Kim J. A study on the mechanical properties and thermal conductivity enhancement through TPU/BN composites by hybrid surface treatment (mechanically and chemically) of boron nitride. Materials Chemistry and Physics 2019;223:607-12.
[189] Yuan F, Jiao WC, Yang F, Liu WB, Xu ZH, Wang RG. Surface modification and magnetic alignment of hexagonal boron nitride nanosheets for highly thermally conductive composites. Rsc Advances 2017;7:43380-9.
[190] Shen WT, Wu W, Liu C, Wang Y, Zhang XW. Thermal conductivity enhancement of PLA/TPU/BN composites by controlling BN distribution and annealing treatment. Plastics Rubber and Composites.
[191] Fei T, Li Y, Liu B, Xia C. Flexible polyurethane/boron nitride composites with enhanced thermal conductivity. High Performance Polymers 2019.
[192] Liu P, Li L, Wang L, Huang T, Yao Y, Xu W. Effects of 2D boron nitride (BN) nanoplates filler on the thermal, electrical, mechanical and dielectric properties of high temperature vulcanized silicone rubber for composite insulators. Journal of

Alloys and Compounds 2019;774:396-404.
[193] Zhong B, Zou J, An L, Ji C, Huang X, Liu W, et al. The effects of the hexagonal boron nitride nanoflake properties on the thermal conductivity of hexagonal boron nitride nanoflake/silicone rubber composites. Composites Part A: Applied Science and Manufacturing 2019;127:105629.
[194] Feng C-P, Wan S-S, Wu W-C, Bai L, Bao R-Y, Liu Z-Y, et al. Electrically insulating, layer structured SiR/GNPs/BN thermal management materials with enhanced thermal conductivity and breakdown voltage. Composites Science and Technology 2018;167:456-62.
[195] Kuo CFJ, Dewangga GRS, Chen JB. Fabrication of a thermally conductive silicone composite by incorporating surface-modified boron nitride. Textile Research Journal 2019;89:2637-47.
[196] Wang JH, Zhao D, Zou X, Mao L, Shi LY. The exfoliation and functionalization of boron nitride nanosheets and their utilization in silicone composites with improved thermal conductivity. Journal of Materials Science-Materials in Electronics 2017;28:12984-94.
[197] Xue Y, Li X, Wang H, Zhao F, Zhang D, Chen Y. Improvement in thermal conductivity of through-plane aligned boron nitride/silicone rubber composites. Materials & Design 2019;165:107580.
[198] Kuo CFJ, Chen JB, Chen PY, Dong MY. Preparation of boron nitride nanosheets using a chemical exfoliation method as a thermal conductive filler for the development of silicone thermal composites Part I: effect of single- and hybrid-filler additions on the silicone composite performance. Textile Research Journal 2020;90:666-84.
[199] Ou Z, Gao F, Zhao H, Dang S, Zhu L. Research on the thermal conductivity and dielectric properties of AlN and BN co-filled addition-cure liquid silicone rubber composites. Rsc Advances 2019;9:28851-6.
[200] Zhao LW, Shi XR, Yin Y, Jiang B, Huang YD. A self-healing silicone/BN composite with efficient healing property and improved thermal conductivities. Composites Science and Technology 2020;186.
[201] Tang XH, Guo YL, Liao ZQ, Fan JH, Zhang K, Yang ZJ, et al. Synergistic enhancement of thermal conductivity between SiCw and h-BN in MVQ-based composite. Fullerenes Nanotubes and Carbon Nanostructures 2019;27:434-9.

[202] 张振昊，赵晓帆，孙海滨. 六方氮化硼合成及其对导热硅橡胶性能的影响. 陶瓷学报 2018;39:244-7.
[203] Ghahramani N, Esfahani SAS, Mehranpour M, Nazockdast H. The effect of filler localization on morphology and thermal conductivity of the polyamide/cyclic olefin copolymer blends filled with boron nitride. Journal of Materials Science 2018;53:16146-59.
[204] Wang L, Zhang LC, Fischer A, Zhong YH, Drummer D, Wu W. Enhanced thermal conductivity and flame retardancy of polyamide 6/flame retardant composites with hexagonal boron nitride. Journal of Polymer Engineering 2018;38:767-74.
[205] Geng Y, He H, Jia Y, Peng X, Li Y. Enhanced through-plane thermal conductivity of polyamide 6 composites with vertical alignment of boron nitride achieved by fused deposition modeling. Polymer Composites 2019;40:3375-82.
[206] Fischer AJ, Zhong YH, Zhang LC, Wu W, Drummer D. Heat propagation in thermally conductive polymers of PA6 and hexagonal boron nitride. Fire and Materials 2019;43:928-35.
[207] Li SZ, Yang TT, Zou HW, Liang M, Chen Y. Enhancement in thermal conductivity and mechanical properties via large-scale fabrication of boron nitride nanosheets. High Performance Polymers 2017;29:315-27.
[208] Fu X, Guo Y, Du Q, Guan L, He S. Improved dielectric stability of epoxy composites with ultralow boron nitride loading. Rsc Advances 2019;9:4344-50.
[209] Yang L, Wang LQ, Chen YH. Solid-state shear milling method to prepare PA12/boron nitride thermal conductive composite powders and their selective laser sintering 3D-printing. Journal of Applied Polymer Science 2020;137.
[210] Wang L, Wu W, Drummer D, Ma RB, Liu ZW, Shen WT. Study on thermal conductive PA6 composites with 3-dimensional structured boron nitride hybrids. Journal of Applied Polymer Science 2019;136.
[211] Shao LB, Shi LY, Li XH, Song N, Ding P. Synergistic effect of BN and graphene nanosheets in 3D framework on the enhancement of thermal conductive properties of polymeric composites. Composites Science and Technology 2016;135:83-91.
[212] 吕昭媛，顾军渭. 静电纺丝-模压法制备氮化硼/聚酰亚胺介电导热复合材料. 2016 年全国高分子材料科学与工程研讨会. 中国广西桂林 2016. p. 1.
[213] 王乐泉，陈英红. 固相剪切碾磨制备 PA12/氮化硼导热复合材料及其选择

性激光烧结 3D 打印研究. 中国化学会 2017 全国高分子学术论文报告会. 中国四川成都 2017. p. 1.
[214] Fang H, Li D, Wu F, Peng X, Chen A, Zhang L, et al. In situ Polymerization of Polyamide 6/Boron Nitride Composites to Enhance Thermal Conductivity and Mechanical Properties
via
Boron Nitride Covalently Grafted Polyamide 6. Polymer Engineering & Science 2020;60:710-6.
[215] Song QS, Zhu W, Deng Y, He DL, Feng JJ. Enhanced thermal conductivity and mechanical property of flexible poly (vinylidene fluoride)/boron nitride/graphite nanoplatelets insulation films with high breakdown strength and reliability. Composites Science and Technology 2018;168:381-7.
[216] Zhang D-L, Zha J-W, Li W-K, Li C-Q, Wang S-J, Wen Y, et al. Enhanced thermal conductivity and mechanical property through boron nitride hot string in polyvinylidene fluoride fibers by electrospinning. Composites Science and Technology 2018;156:1-7.
[217] Teng C, Su LY, Chen JX, Wang JF. Flexible, thermally conductive layered composite films from massively exfoliated boron nitride nanosheets. Composites Part a-Applied Science and Manufacturing 2019;124.
[218] Song Q, Zhu W, Deng Y, Hai F, Wang Y, Guo Z. Enhanced through-plane thermal conductivity and high electrical insulation of flexible composite films with aligned boron nitride for thermal interface material. Composites Part a-Applied Science and Manufacturing 2019;127.
[219] Chen J, Huang X, Sun B, Jiang P. Highly Thermally Conductive Yet Electrically Insulating Polymer/Boron Nitride Nanosheets Nanocomposite Films for Improved Thermal Management Capability. ACS Nano 2019;13:337-45.
[220] Wang Z-G, Huang Y-F, Zhang G-Q, Wang H-Q, Xu J-Z, Lei J, et al. Enhanced Thermal Conductivity of Segregated Poly(vinylidene fluoride) Composites via Forming Hybrid Conductive Network of Boron Nitride and Carbon Nanotubes. Industrial & Engineering Chemistry Research 2018;57:10391-7.
[221] Wang MJ, Jiao ZY, Chen YP, Hou X, Fu L, Wu YM, et al. Enhanced thermal conductivity of poly(vinylidene fluoride)/boron nitride nanosheet composites at low filler content. Composites Part a-Applied Science and Manufacturing 2018;109:321-9.

[222] Zhang J, Liu D, Han Q, Jiang L, Shao H, Tang B, et al. Mechanically stretchable piezoelectric polyvinylidene fluoride (PVDF)/Boron nitride nanosheets (BNNSs) polymer nanocomposites. Composites Part B-Engineering 2019;175.
[223] Du C, Li M, Cao M, Song S, Feng S, Li X, et al. Mussel-Inspired and Magnetic Co-functionalization of Hexagonal Boron Nitride in Poly(vinylidene fluoride) Composites Toward Enhanced Thermal and Mechanical Performance for Heat Exchangers. Acs Applied Materials & Interfaces 2018;10:34674-82.
[224] Zhang X, Zheng J, Iop. Polymer Composites with Enhanced Mechanical and Thermal Properties by Orientating Boron Nitride Flakes. 2nd International Conference on Frontiers of Materials Synthesis and Processing2019.
[225] Qi XD, Wang WY, Xiao YJ, Huang T, Zhang N, Yang JH, et al. Tailoring the hybrid network structure of boron nitride/carbon nanotube to achieve thermally conductive poly(vinylidene fluoride) composites. Composites Communications 2019;13:30-6.
[226] Wang X, Yu ZH, Jiao L, Bian HY, Yang WS, Wu WB, et al. Aerogel Perfusion-Prepared h-BN/CNF Composite Film with Multiple Thermally Conductive Pathways and High Thermal Conductivity. Nanomaterials 2019;9.
[227] Wang Z, Wen YY, Zhao SJ, Zhang W, Ji Y, Zhang SF, et al. Soy protein as a sustainable surfactant to functionalize boron nitride nanosheets and its application for preparing thermally conductive biobased composites. Industrial Crops and Products 2019;137:239-47.
[228] Zeng XL, Sun JJ, Yao YM, Sun R, Xu JB, Wong CP. A Combination of Boron Nitride Nanotubes and Cellulose Nanofibers for the Preparation of a Nanocomposite with High Thermal Conductivity. Acs Nano 2017;11:5167-78.
[229] Hu ZR, Wang S, Chen GK, Zhang Q, Wu K, Shi J, et al. An aqueous-only, green route to exfoliate boron nitride for preparation of high thermal conductive boron nitride nanosheet/cellulose nanofiber flexible film. Composites Science and Technology 2018;168:287-95.
[230] Wu K, Liao P, Du RN, Zhang Q, Chen F, Fu Q. Preparation of a thermally conductive biodegradable cellulose nanofiber/hydroxylated boron nitride nanosheet film: the critical role of edge-hydroxylation. Journal of Materials Chemistry A 2018;6:11863-73.
[231] Chen L, Xiao C, Tang YL, Zhang X, Zheng K, Tian XY. Preparation and properties of boron nitride nanosheets/cellulose nanofiber shear-oriented films with

high thermal conductivity. Ceramics International 2019;45:12965-74.
[232] Wu K, Fang JC, Ma JR, Huang R, Chai SG, Chen F, et al. Achieving a Collapsible, Strong, and Highly Thermally Conductive Film Based on Oriented Functionalized Boron Nitride Nanosheets and Cellulose Nanofiber. Acs Applied Materials & Interfaces 2017;9:30035-45.
[233] Tominaga Y, Sato K, Hotta Y, Shibuya H, Sugie M, Saruyama T. Effect of the addition of Al2O3 and h-BN fillers on the thermal conductivity of a cellulose nanofiber/nanodiamond composite film. Cellulose 2019;26:5281-9.
[234] Li QY, Xue ZH, Zhao JQ, Ao CH, Jia XW, Xia T, et al. Mass production of high thermal conductive boron nitride/nanofibrillated cellulose composite membranes. Chemical Engineering Journal 2020;383.
[235] Sun JJ, Yao YM, Zeng XL, Pan GR, Hu JT, Huang Y, et al. Preparation of Boron Nitride Nanosheet/Nanofibrillated Cellulose Nanocomposites with Ultrahigh Thermal Conductivity via Engineering Interfacial Thermal Resistance. Advanced Materials Interfaces 2017;4.
[236] Bo Z, Ying CY, Zhu HR, Wei X, Yang HC, Yan JH, et al. Bifunctional sandwich structure of vertically-oriented graphenes and boron nitride nanosheets for thermal management of LEDs and Li-ion battery. Applied Thermal Engineering 2019;150:1016-27.
[237] Oh H, Kim J. Fabrication of polymethyl methacrylate composites with silanized boron nitride by in-situ polymerization for high thermal conductivity. Composites Science and Technology 2019;172:153-62.
[238] Morishita T, Takahashi N. Highly thermally conductive and electrically insulating polymer nanocomposites with boron nitride nanosheet/ionic liquid complexes. Rsc Advances 2017;7:36450-9.
[239] Ashton TS, Moore AL. Foam-like hierarchical hexagonal boron nitride as a non-traditional thermal conductivity enhancer for polymer-based composite materials. International Journal of Heat and Mass Transfer 2017;115:273-81.
[240] Liu FH, Li Q, Li ZY, Liu Y, Dong LJ, Xiong CX, et al. Poly(methyl methacrylate)/boron nitride nanocomposites with enhanced energy density as high temperature dielectrics. Composites Science and Technology 2017;142:139-44.
[241] Su C-Y, Wang J-C, Chen C-Y, Chu K, Lin C-K. Spherical Composite Powder by Coupling Polymethyl Methacrylate and Boron Nitride via Spray Drying for Cosmetic Application. Materials 2019;12.

[242] Pullanchiyodan A, Nair KS, Surendran KP. Silver-Decorated Boron Nitride Nanosheets as an Effective Hybrid Filler in PMMA for High-Thermal-Conductivity Electronic Substrates. Acs Omega 2017;2:8825-35.
[243] Tang YL, Xiao C, Ding JW, Hu K, Zheng K, Tian XY. Synergetic enhancement of thermal conductivity in the silica-coated boron nitride (SiO2@BN)/polymethyl methacrylate (PMMA) composites. Colloid and Polymer Science.
[244] Wu X, Yang ZJ, Kuang WY, Tang ZH, Guo BC. Coating polyrhodanine onto boron nitride nanosheets for thermally conductive elastomer composites. Composites Part a-Applied Science and Manufacturing 2017;94:77-85.
[245] Fan Y, Cho UR. Effects of graphite and boron nitride based fillers on mechanical, thermal conductive, and thermo‐physical properties in solution styrene‐butadiene rubber. Polymer Composites 2018;40:E1426-E33.
[246] Cao M, Sun MM, Zhang Z, Xia R, Chen P, Wu B, et al. Effect of the blending processes on selective localization and thermal conductivity of BN in PP/EPDM Co-continuous blends. Polymer Testing 2019;78.
[247] Wu X, Liu W, Zhang C, Ren L. Grafting rubber chains onto boron nitride nanosheets for highly flexible, thermally conductive composites. European Polymer Journal 2018;100:12-7.
[248] Gu J, Meng X, Tang Y, Li Y, Zhuang Q, Kong J. Hexagonal boron nitride/polymethyl-vinyl siloxane rubber dielectric thermally conductive composites with ideal thermal stabilities. Composites Part a-Applied Science and Manufacturing 2017;92:27-32.
[249] Yang D, Ni Y, Kong X, Gao D, Wang Y, Hu T, et al. Mussel-inspired modification of boron nitride for natural rubber composites with high thermal conductivity and low dielectric constant. Composites Science and Technology 2019;177:18-25.
[250] Cao M, Shu JJ, Chen P, Xia R, Yang B, Miao JB, et al. Orientation of boron nitride nanosheets in CM/EPDM Co-continuous blends and their thermal conductive properties. Polymer Testing 2018;69:208-13.
[251] Shu JJ, Xia R, Qian JS, Miao JB, Su LF, Cao M, et al. Preparation and Study on Thermal Conductive Composites of Chlorinated Polyethylene Rubber Reinforced by Boron Nitride Particles. Macromolecular Research 2016;24:640-4.
[252] Wu X, Liu H, Tang ZH, Guo BC. Scalable fabrication of thermally conductive

elastomer/boron nitride nanosheets composites by slurry compounding. Composites Science and Technology 2016;123:179-86.
[253] Xue F, Jin XZ, Xie X, Qi XD, Yang JH, Wang Y. Constructing reduced graphene oxide/boron nitride frameworks in melamine foam towards synthesizing phase change materials applied in thermal management of microelectronic devices. Nanoscale 2019;11:18691-701.
[254] Jia XW, Li QY, Ao CH, Hu R, Xia T, Xue ZH, et al. High thermal conductive shape-stabilized phase change materials of polyethylene glycol/boron nitride@chitosan composites for thermal energy storage. Composites Part a-Applied Science and Manufacturing 2020;129.
[255] Yang J, Tang L-S, Bao R-Y, Bai L, Liu Z-Y, Xie B-H, et al. Hybrid network structure of boron nitride and graphene oxide in shape-stabilized composite phase change materials with enhanced thermal conductivity and light-to-electric energy conversion capability. Solar Energy Materials and Solar Cells 2018;174:56-64.
[256] Yang J, Tang LS, Bao RY, Bai L, Liu ZY, Yang W, et al. An ice-templated assembly strategy to construct graphene oxide/boron nitride hybrid porous scaffolds in phase change materials with enhanced thermal conductivity and shape stability for light-thermal-electric energy conversion. Journal of Materials Chemistry A 2016;4:18841-51.
[257] Yang J, Tang LS, Bao RY, Bai L, Liu ZY, Yang W, et al. Largely enhanced thermal conductivity of poly (ethylene glycol)/boron nitride composite phase change materials for solar-thermal-electric energy conversion and storage with very low content of graphene nanoplatelets. Chemical Engineering Journal 2017;315:481-90.
[258] Yang J, Qi GQ, Tang LS, Bao RY, Bai L, Liu ZY, et al. Novel photodriven composite phase change materials with bioinspired modification of BN for solar-thermal energy conversion and storage. Journal of Materials Chemistry A 2016;4:9625-34.
[259] Jiang Y, Liu YJ, Min P, Sui GX. BN@PPS core-shell structure particles and their 3D segregated architecture composites with high thermal conductivities. Composites Science and Technology 2017;144:63-9.
[260] Kim K, Kim J. BN-MWCNT/PPS core-shell structured composite for high thermal conductivity with electrical insulating via particle coating. Polymer 2016;101:168-75.
[261] Kim K, Kim J. Core-shell structured BN/PPS composite film for high thermal

conductivity with low filler concentration. Composites Science and Technology 2016;134:209-16.
[262] Mosanenzadeh SG, Naguib HE. Effect of filler arrangement and networking of hexagonal boron nitride on the conductivity of new thermal management polymeric composites. Composites Part B-Engineering 2016;85:24-30.
[263] Kim K, Oh H, Kim J. Fabrication of covalently linked exfoliated boron nitride nanosheet/multi-walled carbon nanotube hybrid particles for thermal conductive composite materials. Rsc Advances 2018;8:33506-15.
[264] Ryu S, Kim K, Kim J. Silane surface modification of boron nitride for high thermal conductivity with polyphenylene sulfide via melt mixing method. Polymers for Advanced Technologies 2017;28:1489-94.
[265] Gu J, Guo Y, Yang X, Liang C, Geng W, Tang L, et al. Synergistic improvement of thermal conductivities of polyphenylene sulfide composites filled with boron nitride hybrid fillers. Composites Part a-Applied Science and Manufacturing 2017;95:267-73.
[266] Zhan Y, Ren Y, Wan X, Zhang J, Zhang S. Dielectric thermally conductive and stable poly(arylene ether nitrile) composites filled with silver nanoparticles decorated hexagonal boron nitride. Ceramics International 2018;44:2021-9.
[267] 肖倩. 改性氮化硼/聚芳醚腈导热材料的制备与性能研究 [硕士]: 电子科技大学; 2019.
[268] Zhan Y, Long Z, Wan X, Zhan C, Zhang J, He Y. Enhanced dielectric permittivity and thermal conductivity of hexagonal boron nitride/poly(arylene ether nitrile) composites through magnetic alignment and mussel inspired co-modification. Ceramics International 2017;43:12109-19.
[269] Tu L, Xiao Q, Wei R, Liu X. Fabrication and Enhanced Thermal Conductivity of Boron Nitride and Polyarylene Ether Nitrile Hybrids. Polymers 2019;11.
[270] Xiao Q, Han W, Yang R, You Y, Wei R, Liu X. Mechanical, Dielectric, and Thermal Properties of Polyarylene Ether Nitrile and Boron Nitride Nanosheets Composites. Polymer Composites 2018;39:E1598-E605.
[271] Ryu S, Kim K, Kim J. Silane Surface Treatment of Boron Nitride to Improve the Thermal Conductivity of Polyethylene Naphthalate Requiring High Temperature Molding. Polymer Composites 2018;39:E1692-E700.
[272] Wu K, Li Y, Huang R, Chai S, Chen F, Fu Q. Constructing conductive

multi-walled carbon nanotubes network inside hexagonal boron nitride network in polymer composites for significantly improved dielectric property and thermal conductivity. Composites Science and Technology 2017;151:193-201.
[273] Liu B, Li Y, Fei T, Han S, Xia C, Shan Z, et al. Highly thermally conductive polystyrene/polypropylene/boron nitride composites with 3D segregated structure prepared by solution-mixing and hot-pressing method. Chemical Engineering Journal 2020;385:123829.
[274] Wang R, Cheng H, Gong Y, Wang F, Ding X, Hu R, et al. Highly Thermally Conductive Polymer Composite Originated from Assembly of Boron Nitride at an Oil-Water Interface. ACS Appl Mater Interfaces 2019;11:42818-26.
[275] Wang X, Wu P. Preparation of Highly Thermally Conductive Polymer Composite at Low Filler Content via a Self-Assembly Process between Polystyrene Microspheres and Boron Nitride Nanosheets. Acs Applied Materials & Interfaces 2017;9:19934-44.
[276] 高传伟, 杨昌跃, 陈军, 倪海鹰. 具有相隔离结构的聚苯乙烯/氮化硼绝缘导热复合材料的制备及研究. 塑料工业 2017;45:142-6.
[277] Lin MY, Li YH, Xu K, Ou YH, Su LF, Feng X, et al. Thermally conductive nanostructured, aramid dielectric composite films with boron nitride nanosheets. Composites Science and Technology 2019;175:85-91.
[278] 谢璠, 王亚芳, 卓龙海, 秦盼亮, 宁逗逗, 王丹妮, et al. 高导热氮化硼/芳纶沉析复合薄膜的制备及性能. 高等学校化学学报 2020;41:582-90.
[279] Xiao G, Di J, Li H, Wang J. Highly thermally conductive, ductile biomimetic boron nitride/aramid nanofiber composite film. Composites Science and Technology 2020;189:108021.
[280] Quill TJ, Smith MK, Zhou T, Baioumy MGS, Berenguer JP, Cola BA, et al. Thermal and mechanical properties of 3D printed boron nitride - ABS composites. Applied Composite Materials 2018;25:1205-17.
[281] Li G, Xing R, Geng P, Liu Z, He L, Wang N, et al. Surface modification of boron nitride via poly (dopamine) coating and preparation of acrylonitrile-butadiene-styrene copolymer/boron nitride composites with enhanced thermal conductivity. Polymers for Advanced Technologies 2018;29:337-46.
[282] 邢荣芬. 丙烯腈—丁二烯—苯乙烯共聚物/氮化硼导热复合材料的制备与性能研究 [硕士]: 河北工业大学; 2016.

[283] Sun N, Sun J, Zeng X, Chen P, Qian J, Xia R, et al. Hot-pressing induced orientation of boron nitride in polycarbonate composites with enhanced thermal conductivity. Composites Part A: Applied Science and Manufacturing 2018;110:45-52.
[284] Wang JW, Li HR, Li GH, Liu ZX, Zhang QX, Wang NY, et al. Noncovalent functionalization of boron nitride and its effect on the thermal conductivity of polycarbonate composites. Journal of Applied Polymer Science 2017;134.
[285] 王进炜. 聚碳酸酯/氮化硼导热复合材料的制备与性能研究 [硕士]: 河北工业大学; 2017.
[286] Yang Z, Zhou LH, Luo W, Wan JY, Dai JQ, Han XG, et al. Thermally conductive, dielectric PCM-boron nitride nanosheet composites for efficient electronic system thermal management. Nanoscale 2016;8:19326-33.
[287] Mosanenzadeh SG, Liu MW, Palhares HG, Naguib HE. Design of Thermal Hybrid Composites Based on Liquid Crystal Polymer and Hexagonal Boron Nitride Fiber Network in Polylactide Matrix. Journal of Polymer Science Part B-Polymer Physics 2016;54:457-64.
[288] Mosanenzadeh SG, Khalid S, Cui Y, Naguib HE. High thermally conductive PLA based composites with tailored hybrid network of hexagonal boron nitride and graphene nanoplatelets. Polymer Composites 2016;37:2196-205.
[289] Zhou SS, Xu TL, Jiang F, Song N, Shi LY, Ding P. High thermal conductivity property of polyamide-imide/boron nitride composite films by doping boron nitride quantum dots. Journal of Materials Chemistry C 2019;7:13896-903.
[290] Jiang F, Cui S, Song N, Shi L, Ding P. Hydrogen Bond-Regulated Boron Nitride Network Structures for Improved Thermal Conductive Property of Polyamide-imide Composites. ACS Appl Mater Interfaces 2018;10:16812-21.
[291] Li Z, Kong J, Han L, Zhang H, Dong L. Effect of crystallinity on the thermal conductivity of poly(3-hydroxybutyrate)/BN composites. Polymer Bulletin 2018;75:1651-66.
[292] Li Z, Ju D, Han L, Dong L. Formation of more efficient thermally conductive pathways due to the synergistic effect of boron nitride and alumina in poly(3-hydroxylbutyrate). Thermochimica Acta 2017;652:9-16.
[293] Fink M, Collin D, Loebmann P. Hybrid polymer incorporating BN particles: Thermal, mechanical, and electrical properties. Journal of Sol-Gel Science and

Technology 2017;83:489-94.
[294] Lang K, Loebmann P. BN-hybrid polymer composites: influence of particle surface functionalization. Journal of Sol-Gel Science and Technology 2018;86:135-40.
[295] Zhang XR, Cai XZ, Xie XY, Pu CY, Dong XZ, Jiang ZY, et al. Anisotropic Thermally Conductive Perfluoroalkoxy Composite with Low Dielectric Constant Fabricated by Aligning Boron Nitride Nanosheets via Hot Pressing. Polymers 2019;11.
[296] Wu YP, Xue Y, Qin S, Liu D, Wang XB, Hu X, et al. BN Nanosheet/Polymer Films with Highly Anisotropic Thermal Conductivity for Thermal Management Applications. Acs Applied Materials & Interfaces 2017;9:43163-70.
[297] Lee J, Jung H, Yu S, Cho SM, Tiwari VK, Velusamy DB, et al. Boron Nitride Nanosheets (BNNSs) Chemically Modified by "Grafting-From" Polymerization of Poly(caprolactone) for Thermally Conductive Polymer Composites. Chemistry-an Asian Journal 2016;11:1921-8.
[298] 赵春宝, 徐随春, 赵玮, 张君. BT 树脂/氮化硼导热复合材料的制备与性能研究. 化工新型材料 2018;46:90-3.
[299] Zhang XY, Wang F, Zhu YP, Qi HM. Cyanate ester composites containing surface functionalized BN particles with grafted hyperpolyarylamide exhibiting desirable thermal conductivities and a low dielectric constant. Rsc Advances 2019;9:36424-33.
[300] Goto T, Iida M, Tan H, Liu C, Mayumi K, Maeda R, et al. Development of High Thermally Conductive Flexible Elastomer as a Composite Material of Slide-Ring Material and Plasma-Surface-Modified Boron Nitride Particles: Effect of Plasma-Surface Modification of Boron Nitride Particles. Journal of the Japan Institute of Metals and Materials 2018;82:403-7.
[301] Liu L, Xiao LH, Li M, Zhang XP, Chang YJ, Shang L, et al. Effect of hexagonal boron nitride on high-performance polyether ether ketone composites. Colloid and Polymer Science 2016;294:127-33.
[302] Ge M, Zhang J, Zhao C, Lu C, Du G. Effect of hexagonal boron nitride on the thermal and dielectric properties of polyphenylene ether resin for high-frequency copper clad laminates. Materials & Design 2019;182:108028.
[303] Ye H, Lu T, Xu C, Zhong M, Xu L. Enhanced energy density and thermal

conductivity in poly(fluorovinylidene-co-hexafluoropropylene) nanocomposites incorporated with boron nitride nanosheets exfoliated under assistance of hyperbranched polyethylene. Nanotechnology 2018;29.
[304] Chen L, Li K, Li B, Ren DX, Chen SJ, Xu MZ, et al. Enhanced thermal conductivity of benzoxazine nanocomposites based on non-covalent functionalized hexagonal boron nitride. Composites Science and Technology 2019;182.
[305] Su Z, Wang H, Ye XZ, Tian KH, Huang WQ, Xiao C, et al. Enhanced thermal conductivity of functionalized-graphene/boron nitride flexible laminated composite adhesive via a facile latex approach. Composites Part a-Applied Science and Manufacturing 2017;99:166-75.
[306] Kim D, You M, Seol JH, Ha S, Kim YA. Enhanced Thermal Conductivity of Individual Polymeric Nanofiber Incorporated with Boron Nitride Nanotubes. Journal of Physical Chemistry C 2017;121:7025-9.
[307] Wang Z-G, Liu W, Liu Y-H, Ren Y, Li Y-P, Zhou L, et al. Highly thermal conductive, anisotropically heat-transferred, mechanically flexible composite film by assembly of boron nitride nanosheets for thermal management. Composites Part B: Engineering 2020;180:107569.
[308] Yang YX, Huang LY, Dai QQ, Cui L, Liu SJ, Qi YL, et al. Fabrication of beta-cyclodextrin-crosslinked epoxy polybutadiene/hydroxylated boron nitride nanocomposites with improved mechanical and thermal-conducting properties. Journal of Materials Research and Technology-Jmr&T 2019;8:5853-61.
[309] Tian CF, Yuan L, Liang GZ, Gu AJ. High thermal conductivity and flame-retardant phosphorus-free bismaleimide resin composites based on 3D porous boron nitride framework. Journal of Materials Science 2019;54:7651-64.
[310] Ryu S, Oh H, Kim J. Facile Liquid-Exfoliation Process of Boron Nitride Nanosheets for Thermal Conductive Polyphthalamide Composite. Polymers 2019;11.
[311] Decol M, Pachekoski WM, Becker D. Enhancing thermal conductivity and near-infrared radiation reflectance of poly(epsilon-caprolactone)/poly(lactic acid)-based nanocomposites by incorporating hexagonal boron nitride. Polymer Composites 2019;40:3464-71.
[312] Lin C, Ye X-Y, Xie X-M. High-performance multi-functional graphene/hexagonal boron nitride/poly(ethylene oxide) nanocomposites through enhanced interfacial interaction by coordination. Rsc Advances 2018;8:36761-8.
[313] Li Y, Xu GJ, Guo YQ, Ma TB, Zhong X, Zhang QY, et al. Fabrication, proposed

model and simulation predictions on thermally conductive hybrid cyanate ester composites with boron nitride fillers. Composites Part a-Applied Science and Manufacturing 2018;107:570-8.
[314] Hu K, Yin C, Fan J, Min Y, Xu Q. High-concentration polybenzimidazole-assisted exfoliation of boron nitride nanoflakes for polymer composites. Polymer Composites 2018;39:4285-97.
[315] Jiang H, Wang Z, Geng H, Song X, Zeng H, Zhi C. Highly Flexible and Self-Healable Thermal Interface Material Based on Boron Nitride Nanosheets and a Dual Cross-Linked Hydrogel. ACS Appl Mater Interfaces 2017;9:10078-84.
[316] Goldin N, Dodiuk H, Lewitus D. Enhanced thermal conductivity of photopolymerizable composites using surface modified hexagonal boron nitride fillers. Composites Science and Technology 2017;152:36-45.
[317] Derradji M, Song XM, Dayo AQ, Wang J, Liu WB. Highly filled boron nitride-phthalonitrile nanocomposites for exigent thermally conductive applications. Applied Thermal Engineering 2017;115:630-6.
[318] Morishita T, Okamoto H. Facile Exfoliation and Noncovalent Superacid Functionalization of Boron Nitride Nanosheets and Their Use for Highly Thermally Conductive and Electrically Insulating Polymer Nanocomposites. Acs Applied Materials & Interfaces 2016;8:27064-73.
[319] Yu CP, Gong WB, Zhang J, Lv WB, Tian W, Fan XD, et al. Hot pressing-induced alignment of hexagonal boron nitride in SEBS elastomer for superior thermally conductive composites. Rsc Advances 2018;8:25835-45.
[320] Gu J, Xu S, Zhuang Q, Tang Y, Kong J. Hyperbranched polyborosilazane and boron nitride modified cyanate ester composite with low dielectric loss and desirable thermal conductivity. IEEE Transactions on Dielectrics and Electrical Insulation 2017;24:784-90.
[321] Duan G, Wang Y, Yu J, Zhu J, Hu Z. Improved thermal conductivity and dielectric properties of flexible PMIA composites with modified micro- and nano-sized hexagonal boron nitride. Frontiers of Materials Science 2019;13:64-76.
[322] Chen C, Wang J, Chen X, Yu X, Zhang Q. Improvement of thermal conductivities and mechanical properties for polyphthalonitrile nanocomposites via incorporating functionalized h-BN fillers. High Performance Polymers 2018;31:294-303.
[323] Su Z, Wang H, Ye X, Tian K, Huang W, Guo Y, et al. Non-covalent poly

(2-ethylhexyl acrylate) (P2EHA)/functionalized graphene/h-boron nitride flexible composites with enhanced adhesive and thermal conductivity by a facilitated latex approach. Composites Part a-Applied Science and Manufacturing 2017;99:176-85.
[324] Duan G, Wang Y, Yu J, Zhu J, Hu Z. Novel Poly(m-phenyleneisophthalamide) Dielectric Composites with Enhanced Thermal Conductivity and Breakdown Strength Utilizing Functionalized Boron Nitride Nanosheets. Macromolecular Materials and Engineering 2019.
[325] Su D, Jia YT, Alva G, Tang F, Fang GY. Preparation and thermal properties of n-octadecane/stearic acid eutectic mixtures with hexagonal boron nitride as phase change materials for thermal energy storage. Energy and Buildings 2016;131:35-41.
[326] Shin H, Ahn S, Kim D, Lim JK, Kim CB, Goh M. Recyclable thermoplastic hexagonal boron nitride composites with high thermal conductivity. Composites Part B-Engineering 2019;163:723-9.
[327] Zhao Y, Liu Z, Cao C, Wang C, Fang Y, Huang Y, et al. Self-sacrificed template synthesis of ribbon-like hexagonal boron nitride nano-architectures and their improvement on mechanical and thermal properties of PHA polymer. Scientific Reports 2017;7.
[328] Yi W, Wu W, Drummer D, Shen WT, Chao L, Ning W. Synergistic construction of thermally conductive network in polybenzoxazine with boron nitride hybrid fillers. Materials Research Express 2019;6.
[329] 宋微. 氮化硼@聚苯乙烯类复合材料的制备及导热性质研究 [硕士]: 辽宁大学; 2019.
[330] Goto T, Iida M, Tan H, Liu C, Mayumi K, Maeda R, et al. Thermally conductive tough flexible elastomers as composite of slide-ring materials and surface modified boron nitride particles via plasma in solution. Applied Physics Letters 2018;112.
